# Stimuli-Responsive Membrane Anchor Peptide Nanofoils for Tunable Membrane Association and Lipid Bilayer Fusion

**DOI:** 10.1021/acsami.2c11946

**Published:** 2022-12-06

**Authors:** Vignesh Udyavara Nagaraj, Tünde Juhász, Mayra Quemé-Peña, Imola Cs. Szigyártó, Dóra Bogdán, András Wacha, Judith Mihály, Loránd Románszki, Zoltán Varga, Joakim Andréasson, István Mándity, Tamás Beke-Somfai

**Affiliations:** † Institute of Materials and Environmental Chemistry, Research Centre for Natural Sciences, BudapestH-1117, Hungary; ‡ Hevesy György Ph.D. School of Chemistry, Eötvös Loránd University, BudapestH-1117, Hungary; § Department of Organic Chemistry, Faculty of Pharmacy, Semmelweis University, BudapestH-1092, Hungary; ∥ Department of Chemistry and Chemical Engineering, Physical Chemistry, Chalmers University of Technology, GothenburgSE-412 96, Sweden

**Keywords:** self-assembly, liposomes, membrane activity, spiropyran, peptide bilayer, lipid bilayer fusion

## Abstract

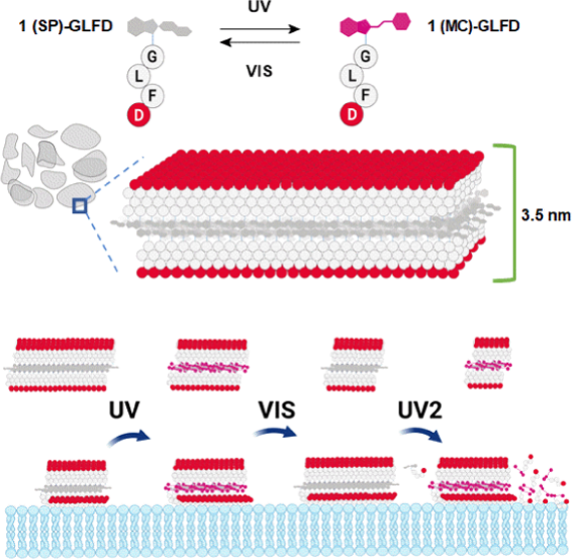

Self-assembled peptide nanostructures with stimuli-responsive features are promising as functional materials. Despite extensive research efforts, water-soluble supramolecular constructs that can interact with lipid membranes in a controllable way are still challenging to achieve. Here, we have employed a short membrane anchor protein motif (**GLFD**) and coupled it to a spiropyran photoswitch. Under physiological conditions, these conjugates assemble into ∼3.5 nm thick, foil-like peptide bilayer morphologies. Photoisomerization from the closed spiro (**SP**) form to the open merocyanine (**MC**) form of the photoswitch triggers rearrangements within the foils. This results in substantial changes in their membrane-binding properties, which also varies sensitively to lipid composition, ranging from reversible nanofoil reformation to stepwise membrane adsorption. The formed peptide layers in the assembly are also able to attach to various liposomes with different surface charges, enabling the fusion of their lipid bilayers. Here, **SP-**to-**MC** conversion can be used both to trigger and to modulate the liposome fusion efficiency.

## 1 Introduction

Bioinspired supramolecular assemblies provide the potential for a wide range of applications in numerous domains of molecular sciences.^1^ Short peptide sequences are particularly interesting as functional materials in bionanotechnology^2,3^ and in biomedicine^4,5^ as their side-chain diversity is accompanied by their prevalent affinity to self-assemble.^6^ Accordingly, numerous ordered structures with beneficial characteristics were achieved,^7−9^ from nanotubes,^10^ through scaffolds inhibiting amyloid aggregation^11^ to membranes.^12^ However, progress with membrane-active supramolecular scaffolds, especially with those that could control vital processes, such as membrane fusion, is still a challenge. By the increased appearance of liposome-encapsulated drugs, such as Caelyx,^13^ controlling dosage may be highly rewarding in reducing side effects. Controlled fusion is also desirable in repairing damaged organelles or cellular integration into complex tissues and organs.^14^ Inversely, the attenuation of specific fusion activity is also important as in the case of viral entry, where inhibitory membrane-active peptides, e.g., in the drug Fuzeon,^15^ demonstrate the potential of peptides interfering with membrane processes. As a natural inspiring example where peptidic self-assembly plays a key role in membrane manipulation, antimicrobial peptides often form temporary, assembled constructs that could be highly relevant for exerting toxicity on targets or for modulating other related biological functions.^16−20^ Recently, it has also been indicated that the manipulation of their membrane activity and thus their antimicrobial affinity can also be achieved by the formation of functional supramolecular coassemblies.^19,21,22^ However, these constructs often adopt only partially ordered assemblies, which limits their controlled use as functional materials.

To match the above delicate goals here, we aimed to identify a suitable shorter peptide sequence capable of incorporating controllable membrane activity. The tetrapeptide sequence **GLFD** is highly conserved in several families of antimicrobial peptides.^23^ In data repository of antimicrobial peptide (DRAMP) database,^24^ various natural peptides comprise **GLFD** motif in common including dahleins, citropins, and aureins. This motif is also present as the N-terminal tail of several proteins such as, e.g., *E. coli* IIA^Glc^ (*Escherichia coli* glucose-specific enzyme IIA), where the N-terminal tail serves as a membrane anchor binding to lipid surfaces.^25^

**Scheme 1:**
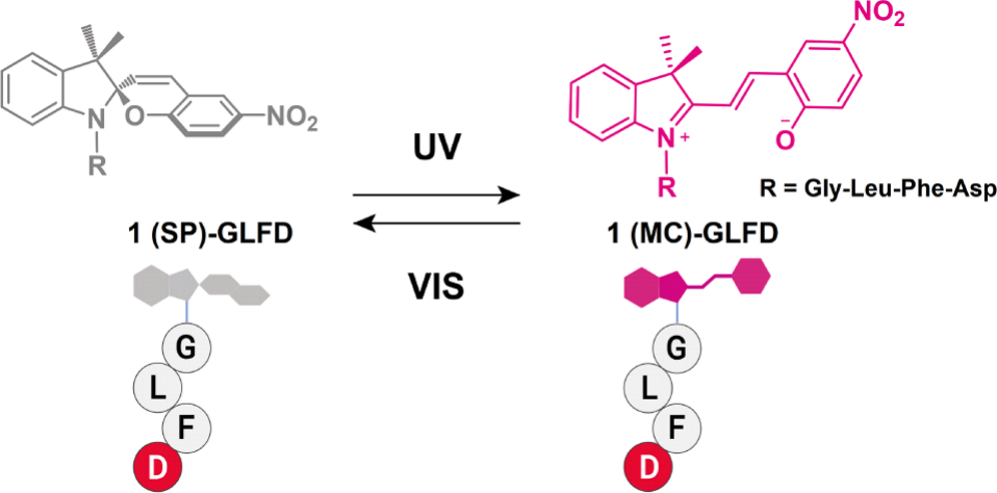
Schematic Description of **1-GLFD** **SP** form (ring closed) and **MC** form (ring opened) of the spiropyran interconverting under UV–vis irradiation (UV λ = 365 nm).

Gaining molecular control over function and activity by external stimuli would open up new and exciting research avenues. Using light as the trigger implies a series of additional advantages, as testified by recent examples of light-controlled protein functionality^26^ and antibiotic activity of small molecules.^27^ In this regard, due to their favorable properties,^28^ photochromic spiropyrans have also demonstrated potential with polymers,^29^ smart material applications,^30^ nanotechnology,^31^ drug delivery,^32^ and biological systems.^33^ Spiropyrans mainly exist in a closed colorless spiro isomer (**SP**) that is dominantly isomerized to the corresponding open-colored merocyanine (**MC**) by UV exposure. The reverse reaction is triggered by exposure to visible light (Scheme 1). Moreover, the relative distribution between these isomeric forms can be affected by solvent polarity, metal ions, acids and bases, temperature, and mechanical force.^34^ Recently, their tendency to form assembled hydrogels^35^ and their membrane-sensitive behavior^36^ has also been described. Recent findings have also shown that spiropyrans can modulate membrane permeability when combined with, e.g., amphiphilic block copolymers.^37−39^

In the present work, we synthesized a tetrapeptide coupled to a spiropyran (**1-GLFD**) and investigated its preparation-sensitive morphologies, membrane activity, and applicability on bringing separate lipid bilayers to proximity for fusion. The interaction of **1-GLFD** with liposomes was studied in both the closed **SP** and the open **MC** forms using UV irradiation and visible light. Results demonstrate that in the presence of coordinating cations, **1-GLFD** readily forms bilayered nanofoil morphologies, where the inner local molecular arrangement can be fine-tuned depending on the preparation mode. Interestingly, in the presence of lipid bilayers, the optical stimulus can induce a transition from a water-soluble form toward membrane adhesion, where this transition process also shows variations depending on the lipid composition.

## 2 Materials and Methods

### 2.1 Peptide Synthesis

Coupling reagents and solvents including 1-[bis(dimethylamino)methylene]-1*H*-1,2,3-triazolo[4,5-*b*]pyridinium-3-oxide hexafluoro-phosphate (HATU), *N*,*N*-diisopropylethylamine (DIPEA), *N*,*N*-dimethylformamide (DMF), 1,8-diazabicyclo[5.4.0]undec-7-ene (DBU), piperidine, and trifluoroacetic acid (TFA) were purchased from Sigma-Aldrich (Budapest, Hungary). The TentaGel R RAM resin was purchased from Rapp Polymere GmbH. SP-GLFD was synthesized by solid-phase technique using continuous flow reactor. TentaGel R RAM resin (0.19 mmol/g) was loaded on the column (125 × 4 mm). Fmoc-protected amino acid (2.5 equiv), 2.5 equiv of HATU as a coupling reagent, and 5 equiv of DIPEA were dissolved in 1.5 mL of DMF. The reaction conditions were 60 bar pressure, 70 °C temperature, and 0.15 mL/min flow rate. Fmoc-deprotection was carried out with the solution mixture containing 2% DBU and 2% piperidine in DMF. Between the coupling cycles, DMF was used for washing. Spiropyran was coupled using the same coupling conditions in DMF and circulated in the column for 24 h at room temperature and atmospheric pressure. Peptide was cleaved from the resin with 95% TFA and 5% water stirring for 3 h. TFA was removed by N_2_ flushing, and the peptide was precipitated in cold diethyl ether. The precipitated peptide was filtered off, dissolved in 10% aqueous acetic acid, and lyophilized. Peptide mass: *m*/*z* calculated for [C_42_H_49_N_7_0_10_] ([M + H]^+^) = 812.36, observed ([M + H]^+^) = 812.60 (Figure S21).

### 2.2 Liposome Preparation

High-purity synthetic DOPC (1,2-dioleoyl-*sn*-glycero-3-phosphocholine), DOPG (1,2-dioleoyl-*sn*-glycero-3-[phospho-rac-(1-glycerol)], sodium salt), and DOTAP (1,2-dioleoyl-3-trimethylammonium-propane, chloride salt) were purchased from NOF Corporation (Tokyo, Japan). The lipid thin film hydration technique was employed to prepare liposomes. Briefly, lipids were dissolved in chloroform (LabScan, Budapest, Hungary) containing 50 vol % of methanol (Reanal, Budapest, Hungary), which were then evaporated by a rotary evaporator. To remove any remaining solvent traces, the lipid film was kept in vacuum for at least 8 h. PBS buffer was used to hydrate the dried lipid film. The solutions after repeated heating (37 °C) and cooling (−196 °C) cycles (at least 10 times) were extruded through polycarbonate filters with a 100 nm pore size (at least 11 times) using a LIPEX extruder (Northern Lipids Inc., Burnaby, Canada). The corresponding lipid stock solution concentration was 13 mM. It was further diluted to 0.635 mM for all of the measurements, which involved liposomes. Pure PC, PG, and 80:20% mixtures of PC/PG and PC/DOTAP were used in the study.

### 2.3 Dynamic Light Scattering and ζ-Potential Measurement

A Litesizer 500 (Anton Paar, Hamburg, Germany) was employed to measure the hydrodynamic diameter and surface charge of the liposomes. The samples were measured using Omega cuvettes. The measurements were performed in automatic mode at 25 °C using a 633 nm He–Ne laser (backscatter detector fixed at 175°, side scatter 90° detector angle, front scatter 15° detector angle). ζ-Potential was also measured under similar conditions, and the data was assessed using the software provided by the manufacturer (see Figures S22–S29 and Tables S3 and S4).

### 2.4 Fluorescence Resonance Energy Transfer

FRET pair (NBD-PE and Rh-PE) was used to monitor liposome fusion. The labeled liposomes were also prepared by the lipid film hydration method, as described above. The labeled liposome contained DOPC (2.54 mM) with 0.8 mol % of NBD-PE (1,2-dioleoyl-*sn*-glycero-3-phosphoethanolamine-*N*-(7-nitro-2-1,3-benzoxadiazol-4-yl)) and Rh-PE (1,2-dioleoyl-*sn*-glycero-3-phosphoethanolamine-*N*-(lissamine rhodamine B sulfonyl)). The labeled and nonlabeled lipids were mixed 1:9 ratio in PBS buffer along with 50 wt % of sucrose and a 1:10 ratio of peptide/lipid. The extent of lipid mixing was estimated using a previous setup as reference^40^ and calculating the relative differences compared to that as follows: *M*(*t*) = 100 × [*I*(s) – *I*(0)]/[*I*(c) – *I*(0)], where *I*(s) is the fluorescence intensity of the samples after rotation, *I*(0) is the fluorescence of the mixture of labeled and unlabeled liposomes before rotation, and *I*(c) is the fluorescence of mock fused liposomes.^40^ The fluorescence intensity spectra were obtained with a Jasco FP-8500 spectrofluorometer. The excitation wavelength was set at 460 nm for NBD-PE/Rh-PE.

### 2.5 Assay Conditions and Preparation of Peptide Nanofoils

The assay buffer used for most of the experiments was isotonic phosphate-buffered saline (PBS, 10 mM phosphate, 137 mM NaCl, 3 mM KCl, pH 7.4), purchased from Sigma-Aldrich (Budapest, Hungary). Low ionic strength tris–HCl buffer (10 mM, pH 7.4) was also used to study the salt effect. For routine experiments, **1-GLFD** powder was dissolved in methanol that was evaporated completely under a vacuum chamber to make a peptide dry film, which was finally hydrated with PBS and sonicated. The two distinct **L** and **S** morphologies were reached by employing 5 and 30 min sonication times, respectively (for more details, see the Supporting Information).

### 2.6 UV–Vis Absorbance Spectroscopy

UV–vis absorbance measurements were carried out in a quartz cuvette with a 1 mm optical path length at 25 °C using a Hewlett–Packard 8453 diode array spectrophotometer. Alternatively, absorbance spectra were obtained by direct conversion of the HT values recorded during CD experiments. Spectra were corrected by subtracting a solvent blank. UV and visible light irradiation were kept for 5 min throughout the experiments.

### 2.7 Circular Dichroism (CD)

A JASCO J-1500 spectropolarimeter was used to collect CD spectra at room temperature in a 0.1 cm path length rectangular quartz cuvette (Hellma, Plainview, NY) in continuous scanning mode between 200 and 600 nm at a rate of 50 nm/min, with a data pitch of 0.5 nm, a response time of 4 s, a 1 nm bandwidth, and 3 times accumulation. The raw spectra were corrected by subtracting a matching blank.

### 2.8 Linear Dichroism (LD)

LD is defined as the differential absorption, ***A***, between the orthogonal forms of the plane polarized light, where the polarization vector of the incident light beam is oriented parallel (***A***_∥_) and perpendicular (***A***_⊥_) to the orientation axis of the sample^41^
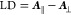


LD is utilized for the systems that are (a) either intrinsically oriented or (b) oriented during the experiment. The sign and amplitude of the LD signal at a given transition are determined by the direction of light passing through the oriented sample. The chromophores will exhibit LD in a macroscopically aligned system if their transition moments have a preferential orientation relative to the system’s orientation axis. The shear flow in a rotation Couette cell device aligns liposomes, resulting in slightly ellipsoidal vesicles. Measurements were carried out on a JASCO J-1500 spectropolarimeter equipped with a Couette flow cell system (CFC-573 Couette cell holder) with a total path length of 0.5 mm. The spectra for all of the samples were recorded between 200 and 600 nm at a rate of 100 nm/min with a data pitch of 0.5 nm, a response time of 1 s, and a 1 nm bandwidth. The baselines at zero shear gradient were measured and subtracted from all spectra. To reduce light scattering, a common approach of refractive index matching, by adding sucrose to the samples, was used.^41^

### 2.9 Atomic Force Microscopy (AFM)

For the atomic force microscope (AFM) imaging and measurements, 1 μL of droplets of 300 μM of **1-GLFD** in PBS solution was placed onto cleaned Si(100) wafer chips and allowed to dry by evaporation in ambient condition at room temperature. Height images were captured in ambient conditions, at room temperature, in tapping mode, in a 512 × 512 pixel resolution using a Dimension 3100 AFM equipped with a NanoScope IIIa controller (Digital Instruments/Veeco). Nanosensors TM PPP-NCHR-20-type silicon cantilevers (thickness: 40 ± 1 μm; length: 125 ± 10 μm; width: 30 ± 7.5 μm; typical resonance frequency of ∼293 kHz; force constant: 10–130 N/m; aluminum-coated top; tip height: 10–15 μm) were used. Raw image data was processed by applying a third-order plane fit followed by a zeroth-order flattening. Cross-sectional height analysis was done by first applying a Gaussian filter (filter size: 0.0380/nm; number of pixels: 9; filter axis: *x*; type: lowpass; cutoff units: spatial freq.) to the processed image in the NanoScope software, then importing it as ASCII into Origin 2018 software, and plotting it as an image with cross sections in Image Profiles mode.

### 2.10 Transmission Electron Microscopy (TEM)

Samples were prepared at a 300 μM peptide in PBS (Figure S20). A droplet (∼5 μL) of the sample was pipetted to a 200-mesh copper grid with a support film made of formvar. After removing the excess liquid, samples were treated/stained with uranyl acetate (2%) followed by drying under an IR lamp. TEM images were obtained by Morgagni 268D (FEI, The Netherlands). Images were captured routinely at magnifications of 11 000×, 28 000×, and 71 000×.

### 2.11 Cryo-Transmission Electron Microscopy (Cryo-TEM)

Four microliters of the sample was applied to freshly plasma-cleaned TEM grids (Quantifoil, Cu, 300mesh, R1.2/1.3) and vitrified into liquid ethane using a ThermoScientific Vitrobot Mark IV (4 °C, 100% rel. humidity, 300 s waiting time, 6 s blotting time). The grids were subsequently mounted into Autogrid cartridges and loaded to Talos Arctica (ThermoScientific) transmission electron microscope for imaging. The microscope was operated at 200 kV. The exosome cryo-TEM micrographs were collected on a Falcon3 direct electron detection camera at a 73 000× nominal magnification with an underfocus of 3 μm and an overall dose of <20 e/Å^2^.

### 2.12 Small-Angle X-ray Scattering (SAXS)

The freshly prepared sample (5 mM) in PBS was promptly filled into a borosilicate glass capillary of ∼1.3 mm outer diameter (<0.01 mm wall thickness) and put into the sample chamber of the CREDO, our in-house SAXS instrument.^42^ Monochromatic Cu Kα (λ = 0.1542 nm) X-rays were generated by a GeniX3D CU ULD integrated beam delivery system (Xenocs SA, Sassenage, France), and the beam was shaped using an optimized three-pinhole collimation scheme.^43^ After interacting with the sample, scattered X-rays were detected by a Pilatus-300k CMOS hybrid pixel 2D position sensitive detector (Dectris Ltd., Baden, Switzerland), placed 528 mm from the sample, the scattering geometry corresponding to the range of 0.2 < *q* < 5 nm^–1^ (*q* being the momentum transfer, defined as *q* = 4π sin θ/λ, where λ is the X-ray wavelength and 2θ is the scattering angle). To control the stability of the instrument and the sample, the exposure was carried out in 12 300 s long parts (corresponding to a total of 1 h of net exposure time), with frequent remeasuring of external and instrumental background signals, as well as calibration samples. After each exposure, the online data reduction routine implemented in the data acquisition software corrected the images for external (background radiation) and instrumental (parasitic scattering from the collimating elements) background noise, sample self-absorption, and detector flatness. The sample-to-detector distance has been calibrated using a mixture of silver behenate and a batch of SBA-15 mesoporous silica with a hexagonal pore structure, precalibrated in-house using first-principles methods. Scattering intensity has been scaled to absolute units (volume-normalized differential scattering cross section) using a piece of glassy carbon, calibrated in turn against water using the method described by Orthaber et al.^44^ Scattering curves were obtained from the fully corrected and calibrated scattering patterns by azimuthal averaging. Exposures affected by excess background radiation were filtered using Tukey’s interquartile range method. The remaining curves were averaged to yield the final scattering curves. The scattering of the solvent (PBS) has been measured and treated under the same conditions and then subtracted from that of the sample. For more details on result analysis, see the Supporting Information.

### 2.13 Infrared Spectroscopy (ATR-FTIR)

A Varian 2000 FTIR Scimitar spectrometer (Varian Inc., Palo Alto, CA) was used for FTIR spectroscopic measurements. The spectrometer is fitted with a liquid nitrogen-cooled mercury–cadmium–telluride (MCT) detector with a “Golden Gate” single reflection diamond ATR accessory (Specac Ltd., Orpington, U.K.). On the diamond ATR surface, 5 μL of the sample was mounted and spectrum was accumulated (2 cm^–1^ resolution and 64 scans) for the dry film after gradual evaporation of the buffered solvent under ambient conditions. ATR correction for every data acquisition, buffer subtraction, and baseline corrections was performed. The GRAMS/32 software package (Galactic Inc.) was used for all spectral manipulations.

### 2.14 NMR Spectroscopy

NMR measurements were carried out at 300K on a Bruker Avance III 500 MHz spectrometer equipped with a cryo probe head. The samples were prepared in 0.5 mL of PBS solutions (H_2_O/D_2_O 90:10 or D_2_O 100% as a solvent) and transferred into 5 mm of NMR sample tubes. For ^1^H NMR measurements, 128 scans, 32k data points, 2.0 s acquisition time, and 6400 Hz sweep width were used. When H_2_O/D_2_O 90:10 was used as a solvent, zgesgp pulse program was used for solvent suppression in the experiments. A mixing time of 300 ms was used for ROESY spinlock. The number of scans was 32, and roesyesgpph pulse sequence was applied. The TOCSY measurement was performed with the mlevesgpph sequence, with a mixing time of 120 ms and the number of scans was 32. For all 2D spectra, 4k time domain points and 512 increments were applied.

### 2.15 Statistical Analysis

All data are shown as the mean ± standard deviation. The mean ± standard deviation for dynamic light scattering and ζ-potential measurement values (Tables S3 and S4) was obtained using the software provided by Anton Paar, Hamburg, Germany. ImageJ software was used to determine the mean diameter, standard deviation, and minimum and maximum sizes of nanofoils based on TEM images (Table S1). The thickness of the nanofoil layers of 3.53 ± 0.09 nm was obtained by SAXS measurements (for calculation, see the Small-Angle X-ray Scattering section of the Supporting Information). An AFM cross-sectional height profile of ∼6.2 nm layer thickness (Figure 2a,c) was analyzed by NanoScope software, then imported it as ASCII into Origin 2018 software, and plotted it as an image with cross sections in Image Profiles mode. UV–vis irradiation was performed three times for absorbance spectroscopy and CD measurements and twice for LD measurements. Each time, two accumulations were acquired during fluorescence measurements.

## 3 Results and Discussion

### 3.1 Self-Assembly in Aqueous Solution

**Figure 1:**
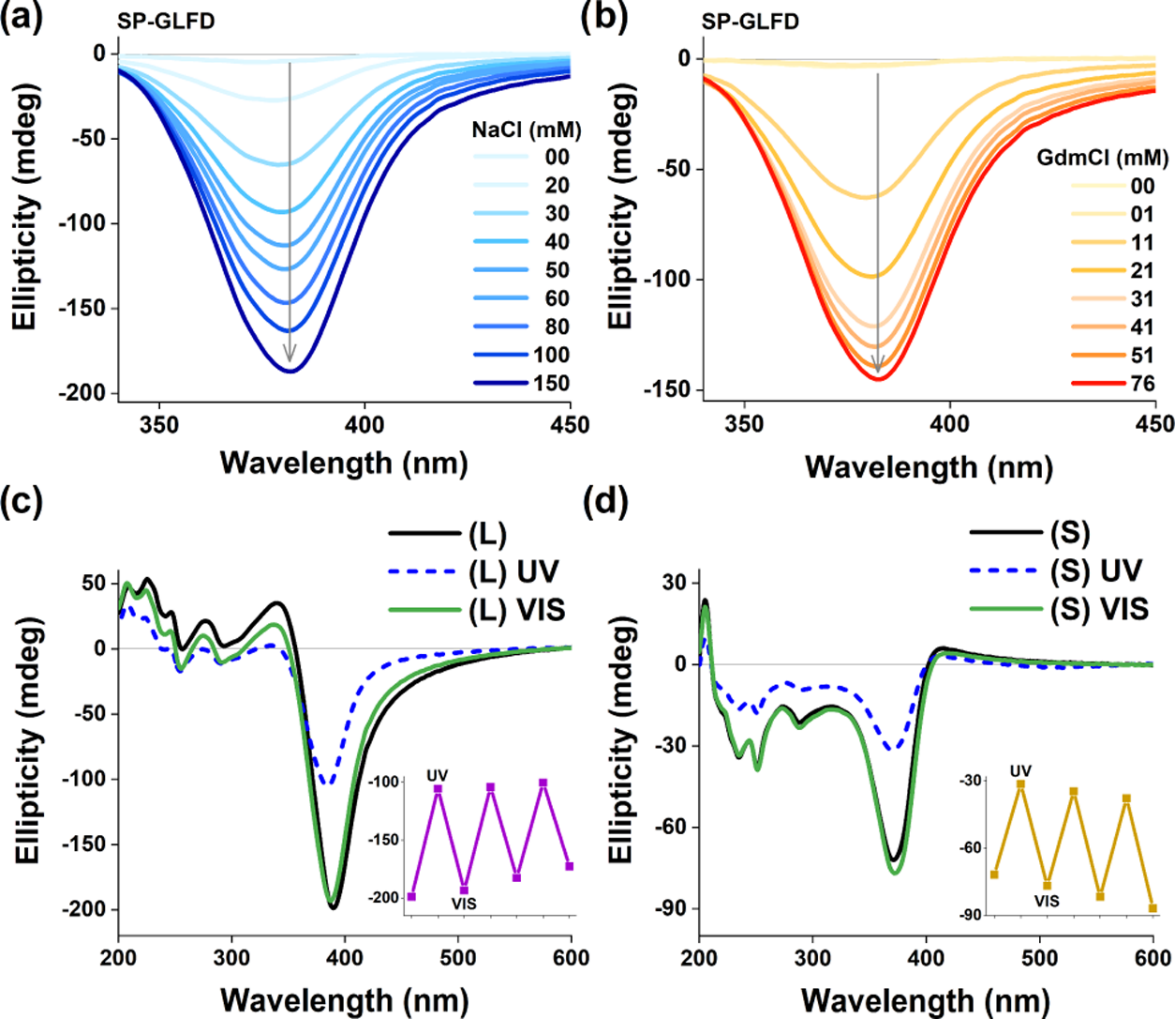
Reversible formation of **1-GLFD** assemblies monitored by the induced CD (ICD) signals. Stepwise addition of (a) NaCl and (b) GdmCl to **1-GLFD** results in a gradual increase of the ICD signal at ∼380 nm. CD spectra of **1-GLFD** (300 μM in PBS buffer) of (c) **L** and (d) **S** forms. The black line displays the CD spectrum of the original morphology. The blue dashed line displays the CD spectrum after irradiation with UV light at 365 nm for 5 min. The green line displays the CD spectrum after subsequent irradiation by visible light for 5 min. Insets: ICD values for several UV–vis cycles indicate reversibility of both morphologies. UV–vis irradiation was repeated three times.

**Figure 2:**
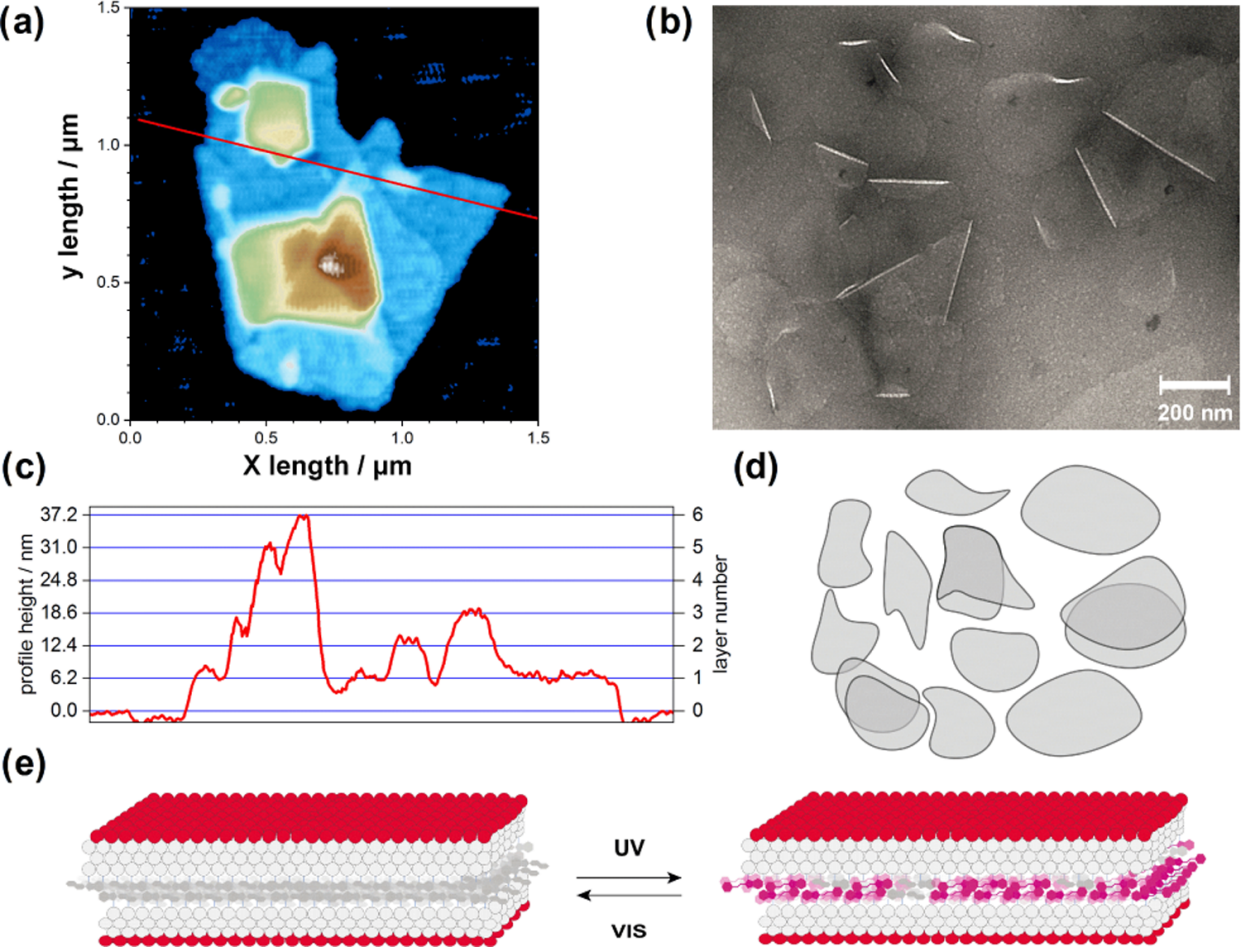
Morphology of **1-GLFD** nanofoils. (a) AFM color-mapped height image of a typical **1-GLFD** assembly on a Si(100) wafer substrate (for details, see the Supporting Information and Figure S5). (b) TEM image of the obtained nanofoils, with normal, overlapping, and partially wrapped-up assemblies, indicating significant flexibility for these systems (for size distribution analysis, see Figure S2 and Table S1). (c) Cross-sectional height profile of the overlapping foils along the red line displayed in panel (a). The height profiles obtained provide an ∼6.2 nm average thickness per layer, which likely includes a hydration shell with sodium ions between the negatively charged foils (for further details, see the Supporting Information). (d) Schematic description of the flexible foils observed in panel (b). (e) Schematic description of the **SP-**to-**MC** conversion depicted within a small section of a nanofoil based on NMR and CD investigations.

The supramolecular morphologies were initially produced by increasing the salt concentration. The process could be monitored by the appearance of an induced CD (**ICD**) signal at ∼380 nm (Figures 1a,b and S1) emerging due to the exciton coupling between the nearby chromene moieties of the spiropyran.^45^ For further preparations, PBS buffer was used that provided sufficient salt concentration to directly reach the supramolecular morphologies by dissolution and sonication. Based on the employed sonication time, two distinct assemblies were identified. Initial structural and morphological investigations demonstrated that shorter and longer sonications resulted in larger (**L**) and smaller assemblies (**S**), respectively, for which the magnitude of ICD values has an apparent linear correlation (Figures 1 and 2). (For more details on sample preparation and assembly size analysis, see Section 2, Figure S2, and Table S1.) Interestingly, upon UV irradiation, the ICD values decrease for both morphologies almost to half (Figure 1c,d). Employing several cycles of UV–vis irradiation, both assemblies display similar reversible spectral characteristics akin to those of previous spiropyran systems,^46,47^ indicating that the **SP** moieties undergo UV-induced ring-opening to the **MC** form and then back to the original **SP** form on exposure to visible light (Figure S3).

### 3.2 Solution-Phase Morphology

NMR spectroscopy studies suggest that within **S** and **L** there are two different arrangements of the individual **1-GLFD** conjugates, and these morphologies display differences as for the photoinduced isomerization processes. For both morphologies, characteristic ^1^H signals were assigned to the **SP** and **MC** forms enabling their identification in the spectra (Figure S4). Initially, the colorless samples contained mainly the **SP** form, 96% for **S** and 85% for **L**. Five minutes of UV irradiation at 365 nm induced a color change from colorless to pink, clearly showing that **SP**-to-**MC** isomerization occurred for both **L** and **S**.

The ratio between **SP** and **MC** forms was, however, rather different. **L** displayed a 77% conversion to **MC**, whereas the conversion for **S** was only 22%. Notably, in both **L** and **S** morphologies, the **MC** form showed excellent thermal stability, as no significant changes in the isomeric distribution **SP**/**MC** were observed after 12 h (72 and 27% for **L** and **S**, respectively). For both samples, the broadening of the signals and low signal intensities in the spectra suggest self-association of the peptides. At the same time, the short **GLFD** peptide motif likely keeps, in part, its dynamic character, which results in very weak correlations in 2D measurements, preventing complete signal assignment and identification of long-range inter-residual NOE-correlations (for more details, see the Supporting Information).

Morphology analysis for **L** and **S** exposed scale- or sheet-like transparent foils (Figure 2), with no considerable apparent difference between the two assembled states. The estimated average size of the overlapping sheets ranged from ∼100 nm to ∼1.5 μm. For **S**, the extended sonication time resulted in more uniform size distribution, between ∼200 and ∼400 nm (mean 198.19 nm, SD 72.60 nm), whereas for **L** the foils are between ∼0.5 and ∼1.5 μm (mean 1.52 μm, SD 0.65 μm) (Figure S2 and Table S1). It can also be observed that these foil morphologies have the tendency to roll up partially or be half-twisted. Measurements by AFM revealed that there can be several layers stacked on top of each other (Figure 2a). Principally, very similar morphologies can be observed in both TEM and cryo-TEM images (Figures 2b and S6). The morphologies were also explored in solution phase by small-angle X-ray scattering (SAXS) measurements, where the thickness of these nanofoil layers was defined to be 3.53 ± 0.09 nm (Figure S7). In line with TEM and AFM data, analysis of the scattering curve indicated that the morphologies had two dimensions, which were much larger than the remaining third one. However, in contrast to dry samples, for solution phase, the absence of diffraction peaks suggested that there was no long-range order of lamellae. Instead, the formed **1-GLFD** nanofoils mainly stood alone.

Considering that the presence of cations is required to reach the assembled morphology (Figure 1), the negatively charged terminal Asp residues are to be positioned on the surface of these constructs, suggesting the formation of a peptide bilayer.^10^ In the case of **1-GLFD**, a bilayer with the spiropyran moieties packed tightly and a fully extended peptide conformation with all dihedrals set to 180° would result in an ∼4.2 nm bilayer width. This length thus suggests that the peptide part is mainly elongated but likely also adopts a conformation that can shield to some extent the more hydrophobic Gly–Leu–Phe part from direct solvent exposure.

### 3.3 Discrimination between **L** and **S** Forms

The **1-GLFD** assembly formation can be most efficiently tracked by CD spectroscopy due to the chiral intermolecular exciton coupling between the respective π–π* transitions of the spiropyran chromophores. ICD signals can arise from both chiral and achiral molecules, including spiro compounds,^48^ where the packing results in a chiral supramolecular assembly such as for H-type or J-type aggregates.^20^ The packing of **1-GLFD** molecules is likely such that the spiropyran units are positioned close to each other. Although the morphology at first seems very similar for **L** and **S**, the NMR investigations could indicate that **S** is more tightly packed, as much smaller amount of **SP** converts to **MC** form for **S** than for **L**. Likewise, while **L** and **S** at first display very similar FTIR spectra, subtle changes in the H-bonding pattern of the peptide backbone amides can be observed (see the Supporting Information). This observation also points toward better-oriented peptide chains in the more tightly packed **S** form. The most distinguishable features that can be used to discriminate between **L** and **S** are the intensity, shape, and position of the ICD signals. The decreased intensity of these peaks upon UV irradiation is likely due to the local rearrangements that follow upon interconversion from the bulky spiropyran moieties to the planar merocyanine isomer, distorting the chiral supramolecular packing of the assembly.^28^ Upon irradiation with visible light, the ICD values are reverted. The reversibility of the isomerization process and the concomitant conformational changes are also supported by FTIR results for both **L** and **S**. While the **L** form showed complete reversibility during two UV/vis cycles for both the ring and peptidic parts, the signals of the **MC** isomer did not appear in the IR spectra of the **S** form until the second cycle. This result is in line with the more tightly packed nature of **S** (for details, see the Supporting Information).

### 3.4 Membrane Behavior and Sensitivity to Lipid Composition

**Figure 3:**
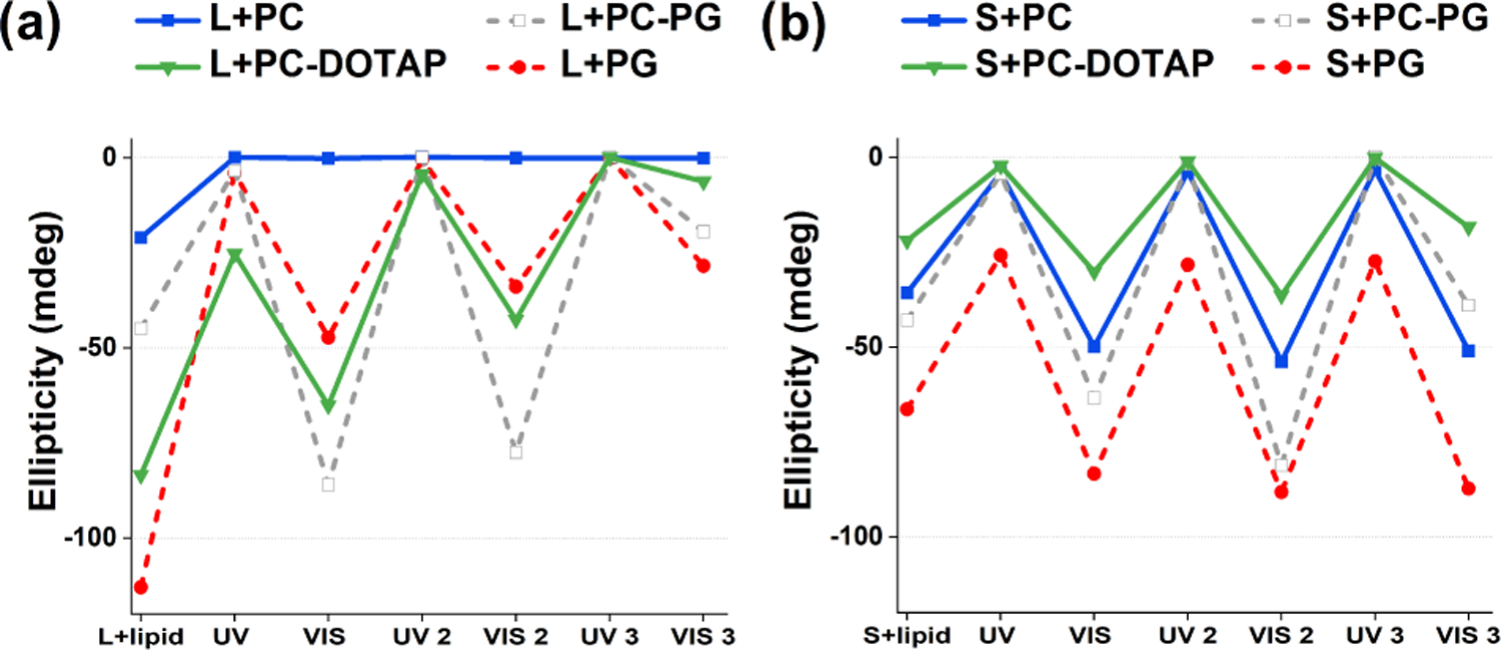
Change of ICD peak intensities upon UV–vis irradiation cycles for (a) **L** and (b) **S** morphologies in the presence of various liposomes. ICD values for neutral PC (100%) (blue, squares) and cationic PC–DOTAP (80:20%) (green, triangles) are displayed by solid lines. Negatively charged PG (100%) (gray, hollow squares) and PC–PG (80:20%) (red, circles) liposomes are displayed by dashed lines. Each irradiation was performed for 5 min, and maximum values of the corresponding ICD peaks are displayed. UV–vis irradiation was repeated three times.

After assessing solution-phase properties, membrane-binding potency was tested for both **L** and **S** morphologies using model vesicles composed of exclusively either zwitterionic PC (1,2-dioleoyl-*sn*-glycero-3-phosphocholine) or the negatively charged PG (1,2-dioleoyl-*sn*-glycero-3-[phospho-rac-(1-glycerol)]). In general, useful insight into morphologic changes in liposomes could be obtained by TEM coupled to freeze fracturing (FF-TEM). However, despite several attempts, the ∼3.5 nm thickness of the nanofoils prevented acquiring useful insight; thus, the stimuli-responsive membrane affinity of the nanofoils was investigated with spectroscopic methods in solution phase. Upon interaction with liposomes, the intrinsic packing differences between **L** and **S** resulted in different behaviors and affinities toward the employed lipid bilayers. Primarily, both **L** and **S** assemblies showed immediate interactions with the liposomes, which resulted in a decreased ICD signal (Figures 3 and S8). When employing the negatively charged PG liposomes, during the UV–vis irradiation cycles, the two systems reacted with similar reversibility as observed for the systems without lipid membranes (Figure S8). **L** and **S**, in principle, retained the reversible changes in the CD signal intensities upon photocycling using UV and visible lights (Figure 3). The latter behavior is likely due to the electrostatic repulsion taking place between the PG lipid bilayer and the **1-GLFD** motif.

When employing the zwitterionic PC liposomes, **S** showed a similar response as for PG, while for **L** the entire ICD peak disappeared after the first UV irradiation and did not reappear upon subsequent vis irradiation. This suggests that the peptide bilayer partially disassembled and **1-GLFD** molecules become membrane-bound. Thus, while **S** could reversibly be reformed by employing photoswitch-cycles, **L** irreversibly transforms into a membrane-bound state. Analysis of lipid vibrations by FTIR spectroscopy in the presence of PC and PG provided similar conclusions (Figure S9a–f). Spectral variations in the lipid C=O band indicated peptide binding mainly affecting the water hydration shell around the lipid neck region. More pronounced lipid perturbations were observed for PC over PG, suggesting higher affinity and likely deeper binding into PC over PG bilayers for both **L** and **S**.

To further assess the membrane-sensitive behavior of the nanofoils, the zwitterionic PC was combined with either the negatively charged PG or with the positively charged DOTAP (1,2-dioleoyl-3-trimethylammonium-propane) (Figure S10). At first, **L** had similar affinity toward PC–PG as to PC, with initial UV irradiation resulting in a decreased ICD value. However, upon exposure to visible light, the large ICD peak reoccurred, suggesting the restoration of the assembled morphology in a reversible manner (Figure 3a). Interestingly, when employing PC–DOTAP, with 20% of the cationic DOTAP lipids, the ICD value for **L** showed a gradual decrease for each irradiation cycle until it almost entirely disappeared by the end of the second cycle (Figure 3a). When considering **S**, neither PC–PG nor PC–DOTAP liposomes had a significant effect on the reversible nature of this morphology during the irradiation cycles tested (Figure 3b). This indicates that the tighter packing in the **S** morphology likely makes the assembled nanofoils more stable and thus less prone to disassembly in the presence of lipid membranes.

The difference in stability between **S** and **L** can be clearly seen when comparing their interactions for the partial negative and partially positive liposomes. The change from 20% negative lipids in PC–PG to 20% positive ones in PC–DOTAP has significant differences for **L** but not for **S**. In a single irradiation cycle, in principle, the relative difference in ICD values seems independent of the liposome used (Figure S10e). However, when using multiple UV–vis cycles (Figure 3), one can observe that for **L**, a marked gradual decrease appears for PC–DOTAP, whereas a more reversible pattern can be seen for PC–PG. This qualitatively suggests that **L** with several cycles can be dissolved into PC–DOTAP but not into PC–PG. In contrast, **S** has the least amount of interactions with PG, and for both PC–PG and PC–DOTAP, it shows a reversible attachment; only the absolute values indicate that for PC–DOTAP, more **1-GLFD** are attached to the surface, potentially in a monomeric form.

Overall, **L** and **S** have distinct affinities for liposomes with different lipid compositions. For PC liposomes, **L** can be transferred into a membrane-associated state that likely results in disassembly of the nanolayer morphology. In the presence of PG or PC–PG liposomes, both **L** and **S** assemblies seem to mostly retain their reversible nature. In contrast, **L** with PC–DOTAP demonstrated a stepwise binding to the lipid bilayer during the irradiation cycles, whereas **S** preserved its reversible nature.

### 3.5 Nanofoil Inner Structure and Membrane-Associated Disassembly

**Figure 4:**
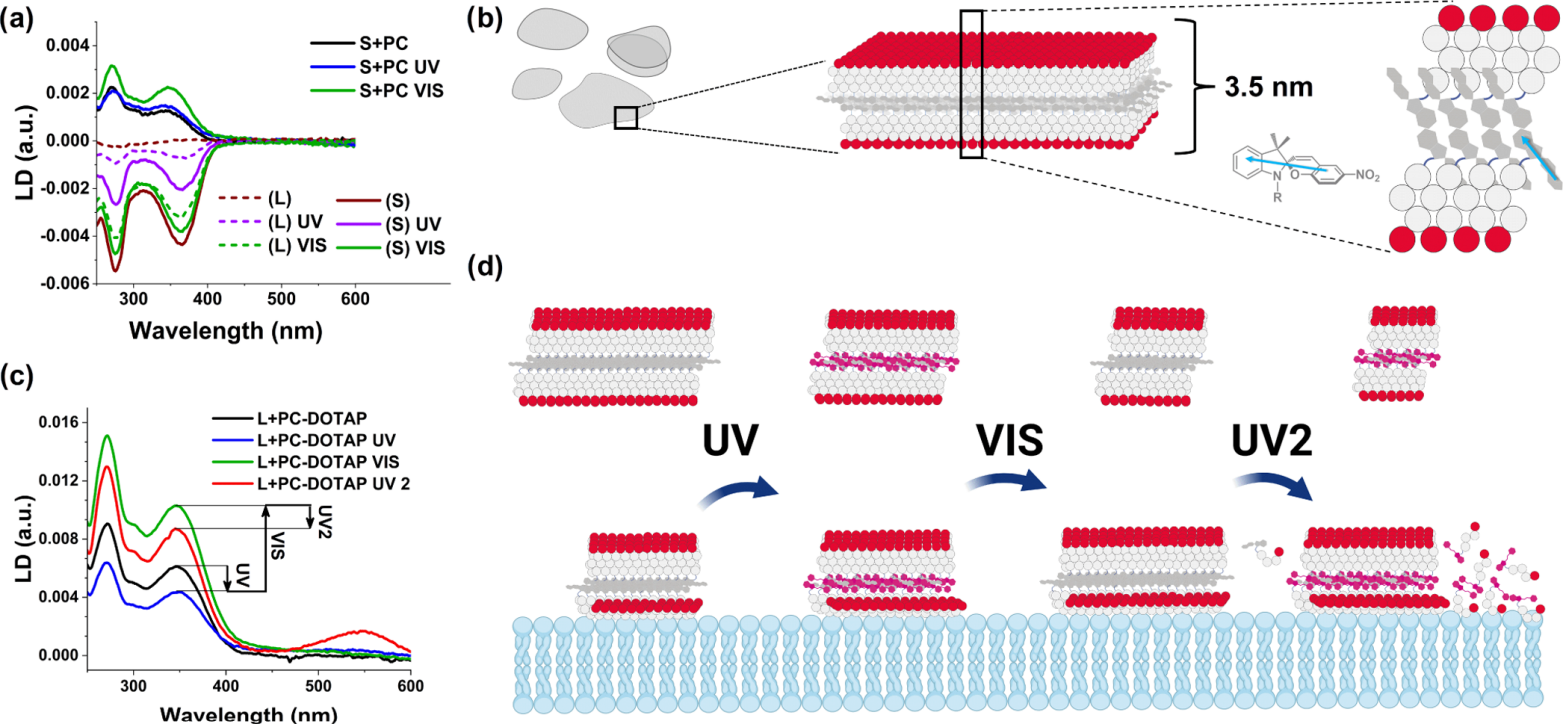
Inner structure and membrane behavior of the formed peptide nanofoils. (a) Flow-LD spectra of the studied **L** and **S** morphologies in solution and in the presence of lipid bilayers. Without liposomes, both **S** (solid lines) and **L** (dashed lines) display negative LD spectra, preserved throughout a UV–vis irradiation cycle. A significant increase in the intensity of the negative LD peaks suggests that the inner orientation of **SP**s within **L** is improved by irradiation. In the presence of liposomes, the LD peaks change the sign for both forms, indicating that the inner orientation of the systems changes significantly when bound to the lipid bilayer surface. (b) Schematic description of the obtained peptide bilayers forming the nanofoils. The direction of the electronic transition dipole moments (TDMs) corresponding to the main peaks at ∼270 and ∼360 nm is displayed as a cyan arrow.^36^ (c) LD spectra of **L** in the presence of PC–DOTAP liposomes during UV–vis irradiation cycles. Note that upon the second UV irradiation (UV2), the appearance of the band at 546 nm indicates the membrane-bound form of individual merocyanine moieties. (d) Schematic mechanism of the stepwise membrane adsorption of **L**, controlled by irradiation steps, to the membrane surface of model liposomes. The estimated relative changes between free and membrane-bound states are displayed on a small subsection of the peptide bilayer. Based on the obtained LD spectra, **1-GLFD** molecules are assumed to become preferentially parallel to the membrane surface when bound to the lipid bilayer (for more details, see the Supporting Information and Figure S19). UV–vis irradiation was repeated two times.

Additional structural insight into the orientation of **SP**s within the nanofoils can be gained by flow-linear dichroism (flow-LD).^36,49,50^ LD is the difference in the absorption of linearly polarized light oriented parallel and perpendicular to a macroscopic orientation axis of the system. In principle, flow-LD uses a very similar setup as the fusion experiments, namely, a shear force is applied in a Couette flow cell, which can render our nanofoils, akin to coin-like bicelles,^49^ oriented.^51^ Flow-LD spectra of **S** in solution showed negative bands at ∼270 and ∼360 nm (Figure 4). Based on previous quantum chemical calculations on the transition dipole moments corresponding to these two bands in the **SP** molecule,^36^ the negative LD signals indicate that in **S** the **SP** chromophores are preferentially oriented parallel to the normal of the nanofoil (Figure 4a,b). This orientation is preserved after a cycle of UV–vis irradiation is employed (for details, see the Supporting Information and Figure S18). For **L**, the initial LD signal is very low, implying a weakly oriented sample (Figure 4a). This is in line with the previous notion of a looser packing for this form. Interestingly, upon employing a UV–vis cycle, the inner **SP** arrangement can be improved, indicating that **L** transforms somewhat toward more tightly packed **S**.

In sharp contrast to the solution-phase LD spectra, in the presence of liposomes, the LD signals are inversed and positive LD peaks can be seen at 270 and 360 nm for all investigated lipid compositions (Figures 4a,c and S19). This suggests that molecules from both **L** and **S** attach to the liposomes, but the inner orientation is altered in the nanofoils. The positive LD signals clearly indicate that the **SP** moieties become predominantly parallel to the surface of the lipid membrane (Figures 4 and S19). This could be due to the rearrangement of the peptidic membrane anchor motif on the lipid surface, where the hydrophobic Gly, Leu, and Phe residues may be assumed to adopt a position parallel to the membrane surface with their apolar regions toward the lipophilic environment. Interestingly, in the case of the cationic PC–DOTAP, the previously mentioned stepwise attachment of **L** to the liposomes can be directly observed (Figures 3a and 4c,d). The intensity of the LD signal changes along the employed irradiation cycles, and during the second cycle, the spectral contribution of the open **MC** form appears as a positive band at 546 nm. This band shows that merocyanine in **1(MC)-GLFD** becomes oriented on the surface of the lipid bilayer, similarly to how the association of **MC** form was observed earlier to countercharged liposomes.^36^

To address whether assembled forms or individual **1-GLFD** monomers interact with the lipid bilayer surfaces, solvatochromic shifts in the absorption maxima of the **MC** form can be informative. In the nanofoil assemblies, the absorption maximum for **1(MC)-GLFD** is ∼520 nm (Table S2). In the presence of liposomes, these values in absorption spectra do not change significantly (∼520–525 nm). This indicates that most of the **1(MC)-GLFD** molecules are still inside the nanofoil morphologies. However, in the presence of PC–DOTAP liposomes after the second UV exposure, the particular LD peak appears at 546 nm, where the wavelength is identical to the peak observed earlier for **MC** alone when it was associated with the surface of a liposome.^36^ This strongly suggests that during the stepwise attachment of **L** to PC–DOTAP, the foils at least partially disassemble and the **1(MC)-GLFD** molecules start to directly interact with the peptide bilayer, most probably as monomers.

### 3.6 Induced Fusion of Lipid Bilayers

A peptide bilayer with membrane-active motifs on both sides, in principle, could be applied to arrange separate liposomes in proximity to each other. To address whether lipid bilayer mixing would ensue, we have chosen to set up a fusion experiment, which relies on mechanical force to drive lipid bilayer fusion.^40^ To test lipid surfaces with different total charges, the PG, PC, and PC–DOTAP liposomes were selected and put in a crowding environment placed in a Couette flow cell where the sample is confined between two rotating cylinders producing shear force in the sample.^40,51^

**Figure 5:**
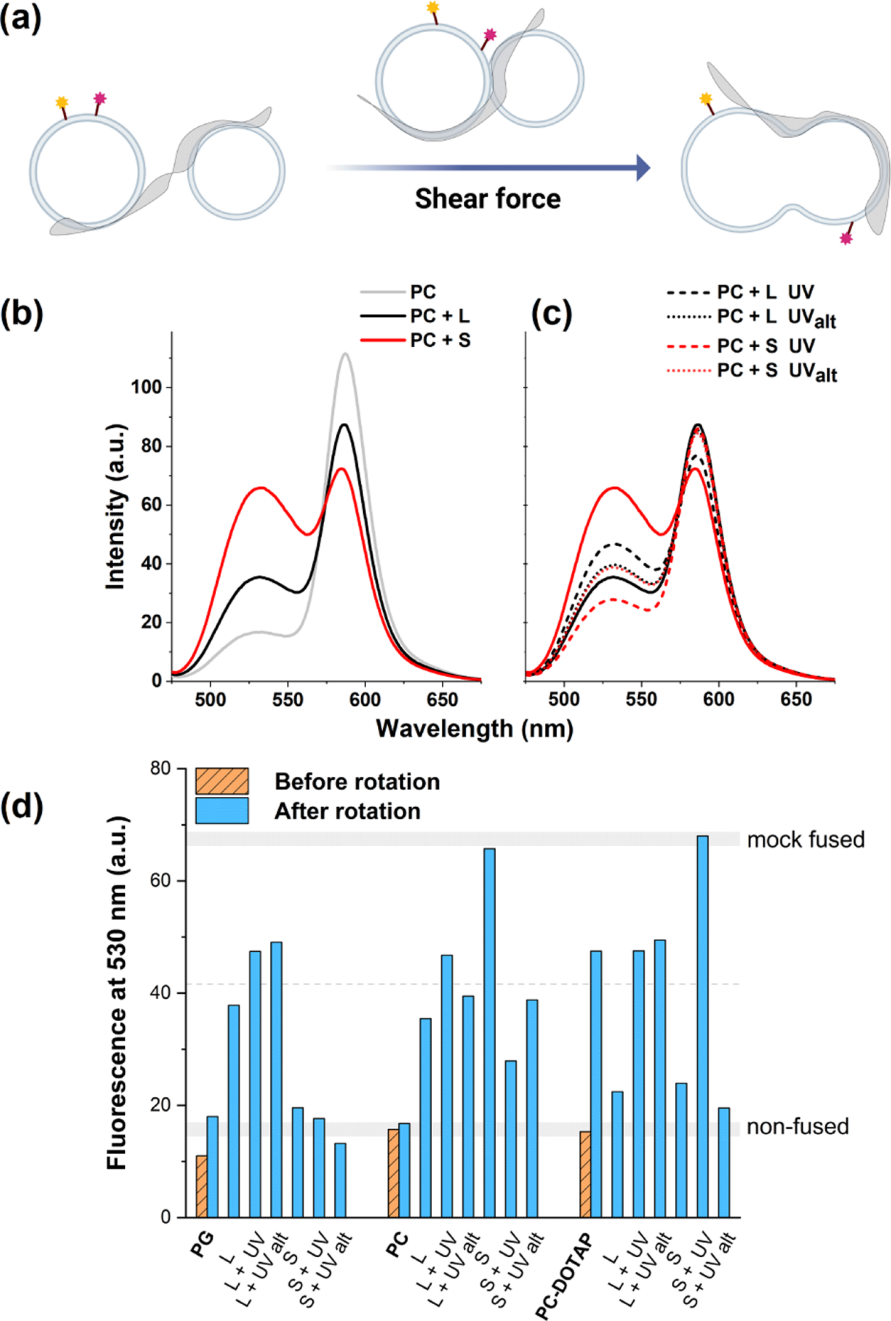
Nanofoil-assisted lipid bilayer mixing. (a) Schematic depiction of a plausible lipid membrane fusion process in the presence of nanofoils under applied shear force. Labeled lipids are highlighted by yellow and pink stars, outlining the lipid mixing observed during the employed FRET assays. (b, c) FRET efficiency of **L** and **S** nanofoils with labeled (NBD-PE/Rh-PE/PC) and nonlabeled (PC) liposomes in a 50 wt % sucrose buffer. The samples were rotated for 20 min with a shear rate of 3100 s^–1^. As a control, labeled liposomes were mixed with nonlabeled liposomes; **L** and **S** were added to control and shear flow was applied as above. All spectra were recorded using two accumulations. A single UV irradiation was applied prior to rotation for **L** and **S**. For UV_alt_ irradiation was applied for **L** and **S** prior to adding sucrose to the samples. (d) Bar graph representing the fluorescence intensities of controls (labeled and nonlabeled liposomes) with **L** and **S**, followed by UV and UV_alt_ irradiations (see Figures S11–S17 for before and after shearing with rotation of respective samples).

In this setup, the *in vivo* crowding is modeled by the addition of sucrose.^52,53^ Mixing of separate lipid bilayers was monitored by exploiting fluorescence resonance energy transfer (FRET) using liposomes incorporating the standard FRET pairs NBD-PE (1,2-dioleoyl-*sn*-glycero-3-phosphoethanolamine-*N*-(7-nitro-2-1,3-benzoxadiazol-4-yl)) and Rh-PE (1,2-dioleoyl-*sn*-glycero-3-phosphoethanolamine-*N*-(lissamine rhodamine B sulfonyl)) (Figure 5). Previously, it has been shown that in this viscous solution lipid bilayer fusion occurred under shear flow at higher shear rates (∼6200 s^–1^) in 1 h, where concomitant lipid mixing could be tracked by the decrease in the efficiency of FRET, leading to increased and decreased fluorescence intensities of NBD and Rh, respectively.^40^ Here, we set lower shear flow and a shorter 20 min rotation time to distinguish easily between the fusion efficiency of the different setups.

Initial experiments were performed on PC liposomes. When using **S** and **L**, results indicated that even the application of a lower, ∼3100 s^–1^, shear rate resulted in liposome fusion compared to the control with PC vesicles alone (Figures 5 and S11). For **S**, this relatively short time resulted in nearly complete lipid mixing, ∼80%, when compared to fluorescence intensity ratios of mock fused liposomes (for details, see Section 2).^40^ For **L**, only limited lipid bilayer mixing occurred (∼20%), whereas for the control, the low shear flow and short mixing time resulted in no significant fusion compared to the initial stage (Figures S12 and S13). Interestingly, when applying a single UV irradiation, we see an opposing effect for **S** and **L**. While for **S** UV irradiation decreased the rate of lipid mixing (from ∼80 to ∼20%), for **L** the single irradiation has somewhat increased it (Figure 5c,d). Furthermore, when UV irradiation was initiated before adding sucrose during sample preparation (UV_alt_), the change in peak intensities was again different, rather similar to the extent observed for **L**, but without UV light stimulus (Figure 5c). As a general conclusion, for PC liposomes, these nanofoils enable fusion pathways in every setup tested, irrespective of the irradiation state.

The same experiments were also performed for PG and PC–DOTAP liposomes (Figure 5d). For PG liposomes, **L** and **S** primarily have distinct effects on fusion affinity. The presence of **L** induces the fusion of PG liposomes, and both types of UV irradiations could further increase the rate of lipid mixing (Figure 5d). Inversely, **S** has apparently no significant influence on the lipid bilayer mixing of the negatively charged PG liposomes and this applies to the UV-irradiated states as well. In the PC–DOTAP system, the lipid bilayer mixing occurs under shear force without the presence of peptide nanofoils. This is in line with previous observations on DOTAP-containing liposomes, where the cationic lipid that has a smaller headgroup region compared to that of phosphatidylcholine lipids can lead to fusogenic properties for liposomes.^54,55^ Here, the presence of both **L** and **S** seems to practically stop the fusion process in the same setup. However, for three out of the four samples, which were exposed to UV light, the fusion of PC–DOTAP systems is undisturbed. For the two UV-irradiated **L** systems, the ratio of lipid mixing becomes comparable to that of the original liposomes, while for **S** + UV this results in a near-complete lipid mixing (Figure 5d). For the three lipid systems tested, the fusion of the lipid bilayers could also be tracked on the trends for DLS measurements performed on all samples before and after rotation, where a qualitative overall increase in the particle sizes could be seen after rotation (data not shown).

From a general perspective, it is particularly exciting that by combining the two morphologies and altering UV irradiation, different mixing efficiencies can be achieved, which shows also some variations depending on the composition of the employed liposomes. For the negatively charged PG liposomes, only **L** induces significantly increased lipid mixing (Figure 5d). This is likely the effect of the potentially looser inner composition of **L**, for which UV light can isomerize the **SP** form into the open **MC** form in a high percentage as described above. For **S**, the lack of induced lipid mixing is likely the consequence of its observed low binding affinity for PG (Figure 3). For the neutral PC liposomes, all six types of setups induced liposomes fusion but to a different extent. For **L**, the two irradiation methods further increased its ratio, whereas for **S** the UV-irradiated versions resulted in a reduced affinity. For the positively charged PC–DOTAP, the spontaneous lipid mixing is attenuated by the nanofoils. However, for all samples, UV light stimulus seems to restore the original fusogenic affinity of PC–DOTAP liposomes, except for **S** + UV_alt_.

Out of the 18 samples with nanofoils, 13 had a marked effect on the lipid mixing affinity of the original liposomes (Figure 5d). UV irradiation of the peptide nanofoils increased (or restored for PC–DOTAP) lipid mixing rate for 9 out of 10 samples compared to that of the original liposomes (the inactive **S** system for PG is excluded). When considering the timing of UV irradiation, mixed results can be observed (Figure 5d). For some of the samples when UV light was applied prior to sucrose addition, we observed higher lipid mixing efficiency compared to UV irradiation when sucrose was already present in the samples, but for others, the effects were inverse. A plausible explanation is that this subtle difference originates from solute cavities appearing in the viscous sucrose solutions.^53^

From an application point of view, it seems particularly useful that (1) **S** is selective for neutral and positively charged liposomes, (2) both **L** and **S** can withhold fusion for DOTAP-containing liposomes, where fusion can be then retriggered by UV light stimulus, and (3) for neutral liposomes, the rate of lipid mixing can be fine-tuned on purpose by the different setups investigated. The above experiments extrapolate that the current tetrapeptide conjugated with a spiropyran can lead to nanofoils, which can interact with more sophisticated lipid compositions to aid and regulate biomembrane fusion processes at a desired rate. The observations suggest widespread applicability of **1-GLFD** and new, potentially similar **SP** conjugates of short peptides. Overall, based on the differences observed for **S** and **L** morphologies, in the presence of lipid membranes, the two morphologies have different stabilities. It is likely that **L** and most of the UV-irradiated samples will contain larger amounts of partially disassembled foils, and the **1-GLFD** molecules could also individually interact with the vesicle surfaces. This process seems to increase the lipid mixing rate in most of the experiments performed as detailed above.

When jointly considering all of the above results, and also the fact that **1-GLFD** with its negatively charged C-terminal aspartate forms nanolayers only in the presence of cations, it is concluded that the nanofoils are most likely peptide bilayers, where the negatively charged Asp residues are on the surface of these (Figures 2 and 4). The formed morphologies are very similar for **L** and **S**, as seen on TEM and AFM images. The layer thickness of 3.53 nm in solution is very close to the theoretical 4.2 nm of two layers of **1-GLFD** molecules in fully extended conformations and is also in line with the previously observed similar peptide layers.^10,12^ ICD signals, NMR results, and LD spectra jointly suggest that within these peptide bilayers the hydrophobic **SP** moieties are closely packed to each other, oriented preferentially parallel to the nanofoil normal. Note that during initial sample preparations **L** can be irreversibly transferred to **S** as detailed above, resulting in a tighter packing and less efficient UV-induced isomerization of **SP** → **MC**. This observation is in full agreement with the NMR and the LD investigations of the two forms after irradiation. Most importantly, the photoswitch moiety offers control of the inner structure of the peptide bilayers, which in turn also affects the nanofoil formation. This can particularly be observed in the presence of lipid membranes with different compositions and also in the fusion experiments performed, where likely several populations coexist such as water-soluble foils, membrane-associated assemblies, and monomeric **1-GLFD** molecules in different ratios depending on the lipid compositions. The combined results suggest that the **SP** → **MC** isomerization can result either in the simultaneous disassembly and irreversible membrane insertion of the peptides to the lipid bilayers (**L** with PC liposomes) or in a stepwise membrane association through several cycles (e.g., **L** with PC–DOTAP).

Excitingly, most of the nanofoils can efficiently induce liposome fusion in a crowded environment mimicking *in vivo* intracellular milieu. This fusion efficiency can be greatly tuned by various isomerization schemes. This demonstrates that the nanofoils will contribute to bringing the vesicles close to each other (Figure 5a) resulting in increased lipid bilayer mixing. Inversely, the presence of the nanofoils may also tether fusogenic DOTAP vesicles to prevent their fusion, but upon irradiation they can re-establish their lipid mixing rates, thereby producing a system with potentially triggerable, controlled fusion properties. Finally, in the presence of negatively charged lipid components, the formed layers retain their reversible nature to a larger extent, most likely due to the electrostatic repulsion occurring between the surfaces of the peptide and the membrane bilayers.

## 4 Conclusions

The short membrane anchor motif **GLFD** has been conjugated to a spiropyran molecular photoswitch. This yielded a peptide bilayer system that has the capacity both for nanofoil formation in aqueous solution and for strong association with lipid membranes. These two states are in a delicate balance, where the equilibrium can be conveniently controlled by isomerization between the **SP** and **MC** forms of the spiropyran. For **L**, a varied response was found to PC, PG, PC–PG, and PC–DOTAP liposomes suggesting sensitive behavior depending on lipid compositions. In contrast, the more tightly packed **S** may grant a more stable nanofoil morphology, which is less sensitive to subtle environmental changes. The bilayer form of the peptidic membrane anchor motifs enabled the mixing of lipid bilayers from separate liposomes. The membrane fusion efficiency in these studies could be fine-tuned by the employed morphologies and the varied timing of the spiropyran photoisomerization. From a practical aspect, the induced CD signals offer a quick and straightforward way to distinguish between the various forms and states of the system. Similar photoswitchable membrane-active peptide systems will likely aid the development of diverse applications. In this regard, the manipulation of biological vesicles or delivery of lipophilic molecules would all be steps toward the practical exploitation of controllable lipid bilayer fusion, directions currently investigated in our laboratory.

## Supporting Information Available

Additional experimental data including supporting figures and supporting texts on infrared analysis and NMR data (PDF)

## Notes

All of the findings of this study and supporting data are available from the authors upon request.

## Abbreviations

DOTAP: dioleoyl-trimethylammonium-propane
GdmCl: guanidinium chloride
MC: merocyanine
PC: 1,2-dioleoyl-*sn*-glycero-3-phosphocholine
PG: 1,2-dioleoyl-*sn*-glycero-3-[phosphor-rac-(1-glycerol)]
SP: spiropyran
NBD-PE: 1,2-dioleoyl-*sn*-glycero-3-phosphoethanolamine-*N*-(7-nitro-2-1,3-benzoxadiazol-4-yl)
Rh-PE: 1,2-dioleoyl-*sn*-glycero-3-phosphoethanolamine-*N*-(lissamine rhodamine B sulfonyl)
