# Supplementary material for: Stimuli-Responsive Membrane Anchor Peptide Nanofoils for Tunable Membrane Association and Lipid Bilayer Fusion
Source: ACS Appl Mater Interfaces. 2022 Dec 6;14(50):55320–31. doi: 10.1021/acsami.2c11946 (PMC9782321; doi:10.1021/acsami.2c11946)
Supplement: Supplementary file 1 — am2c11946_si_001.pdf [file am2c11946_si_001.pdf]

Stimuli-responsive membrane anchor peptide nanofoils for tunable membrane association and lipid bilayer fusion

Vignesh Udyavara Nagaraj, Tünde Juhász, Mayra Quemé-Peña, Imola Cs. Szigyártó, Dóra Bogdán, András Wacha, Judith Mihály, Loránd Románszki, Zoltán Varga, Joakim Andréasson, István Mándity, and Tamás Beke-Somfai*

*Corresponding author address:

[1]Institute of Materials and Environmental Chemistry, Research Centre for Natural Sciences, Budapest H-1117, Hungary

[2]Department of Chemistry and Chemical Engineering, Physical Chemistry, Chalmers University of Technology, Gothenburg SE-412 96, Sweden

E-mail: beke-somfai.tamas@ttk.hu

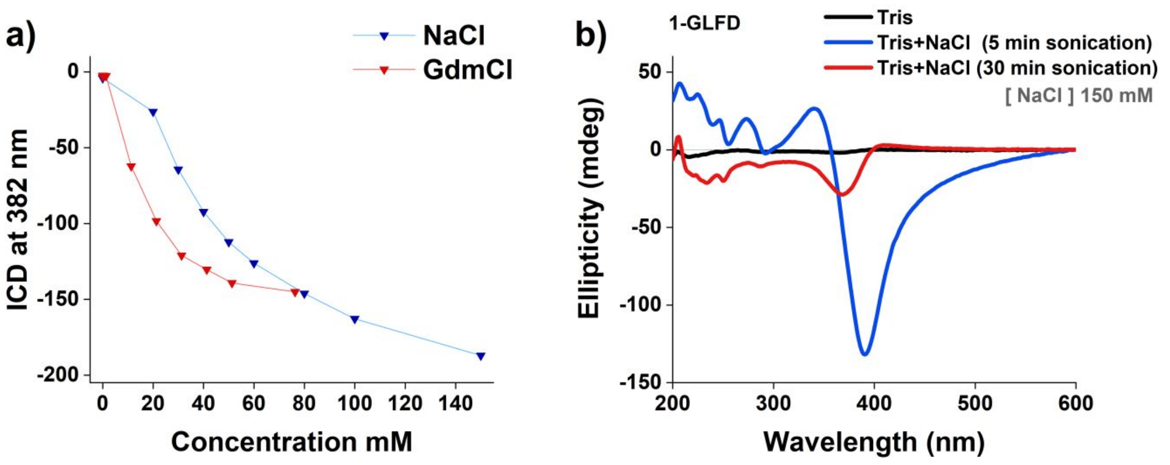

Figure S1. a) Titration of **1-GLFD** with NaCl and guanidinium chloride (GdmCl). b) CD spectra of **1-GLFD** (300 μM) dissolved in Tris prior or after addition of 150 mM NaCl, after a brief (5min) or long (30 min) sonication. Note that the ICD signal reduced significantly upon longer sonication, likewise as observed in PBS.

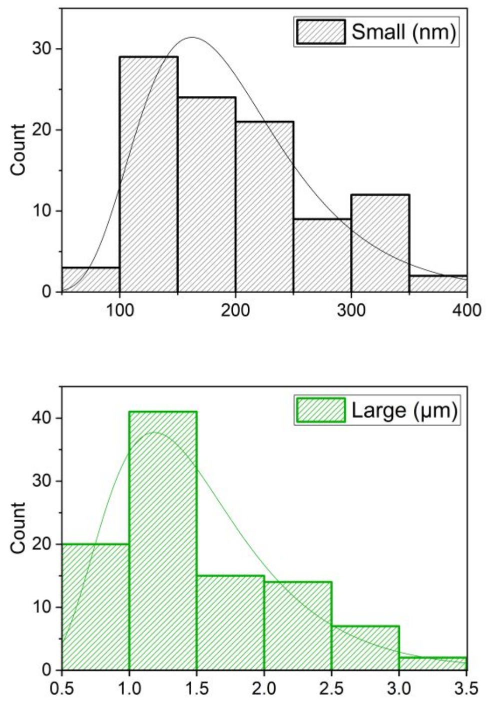

Figure S2. Particle size distribution of **L** and **S** nanofoils from TEM imaging determined using ImageJ software . Data are based by measuring 100 individual nanofoils. (For mean diameter values see Table S1) .

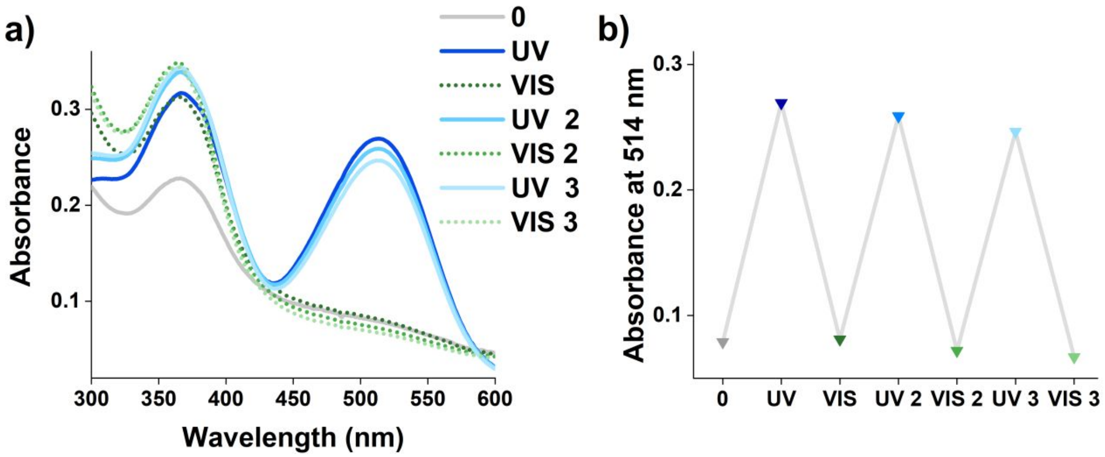

Figure S3. Reversibility of ring opening and closing of **1-GLFD**. a) Absorbance spectra of **1-GLFD** (300 μM) in PBS upon UV-vis irradiation cycles b) and the corresponding peak intensity changes at ~514 nm . In aqueous solution, the spiropyran moiety of the **1-GLFD** peptide shows an absorbance peak at ~365 nm that corresponds to the closed **SP** form **1(SP)-GLFD** and after UV irradiation (λ = 365 nm) has an emerging characteristic signal at ~514 nm that belongs to the open **MC** form, **1(MC)-GLFD**. The switching between the closed and open forms is stable for the cycles investigated.

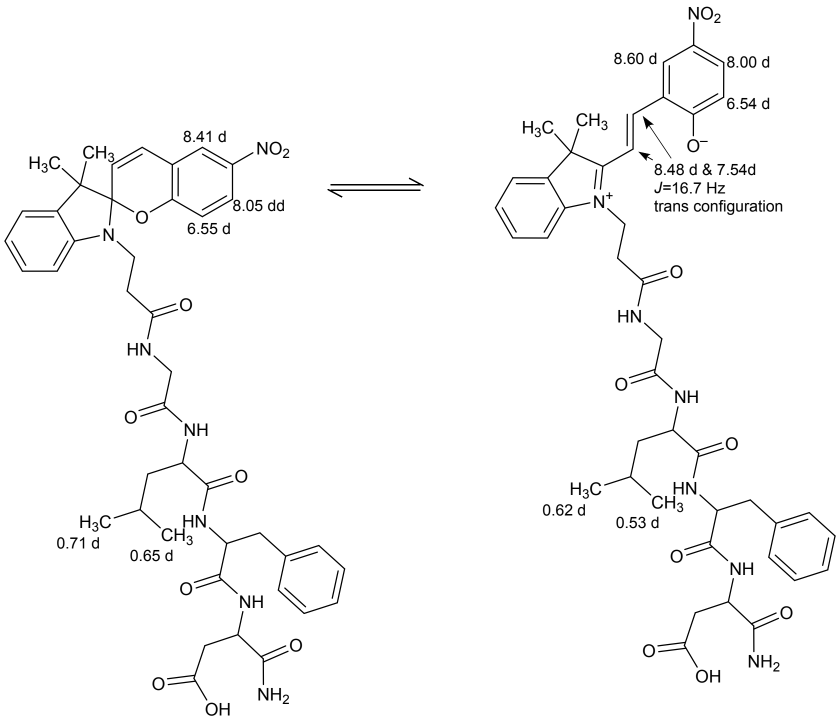

Figure S4. Characteristic 1H NMR signal assignment of the SP (left)and MC (right) forms. For more details see SI text on NMR spectroscopy.

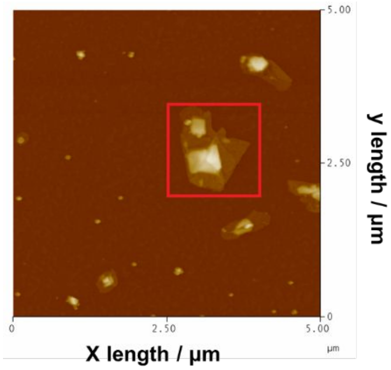

Figure S5. A representative AFM height image of typical **1-GLFD** assemblies on Si (100) wafer substrate.

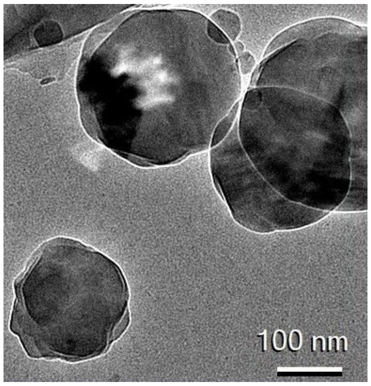

Figure S6. Cryo-transmission electron microscopy image of **1-GLFD** nanofoils.

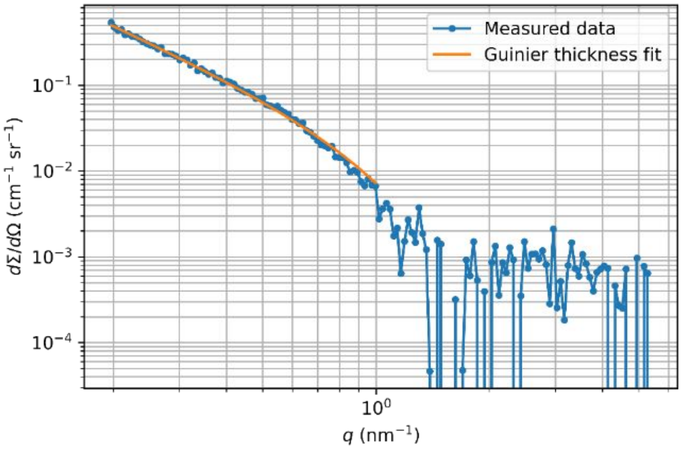

Figure S7. Background-subtracted scattering curve of **1-GLFD**. For more details see SI text on SAXS.

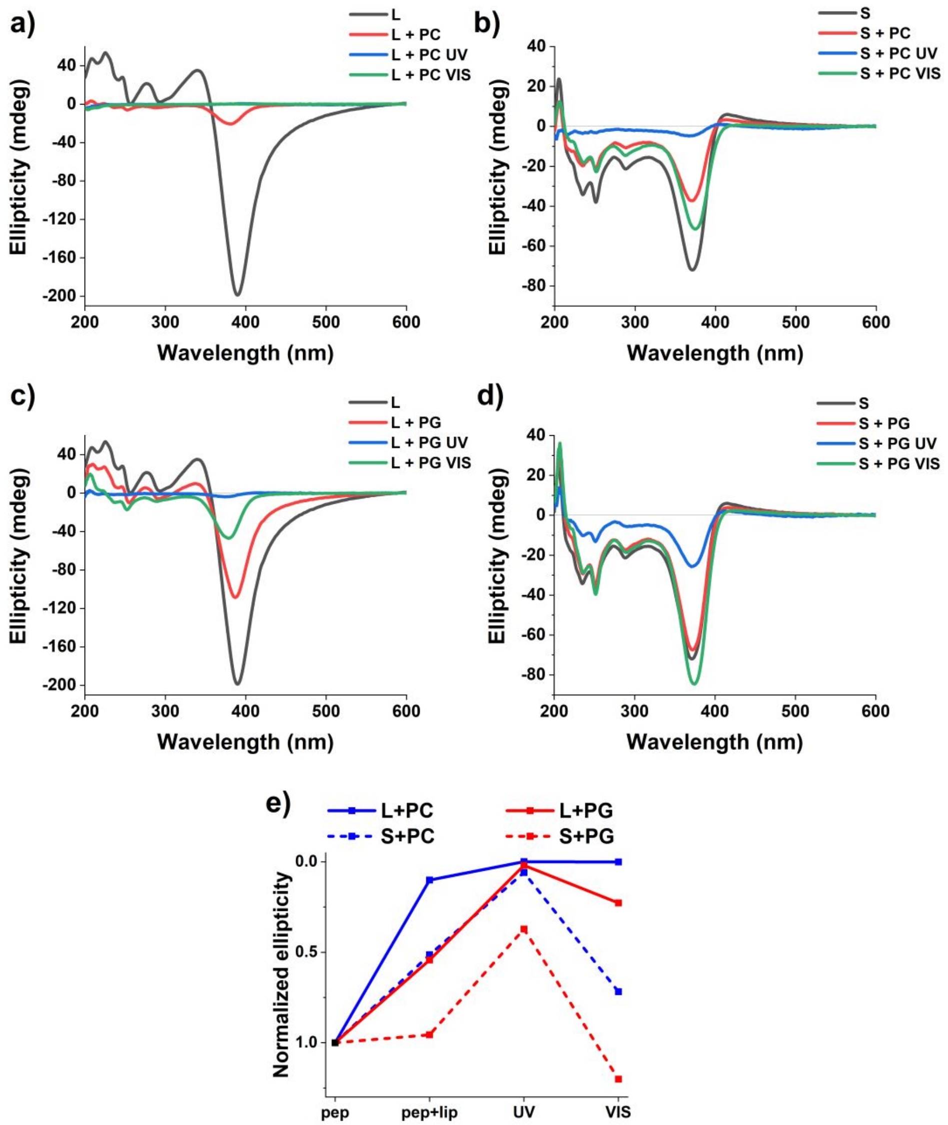

Figure S8. CD spectra of **L** and **S** with PC and PG liposomes, and upon subsequent UV-vis irradiation. Spectra were recorded at 300 μM peptide and 0.635 mM lipid. Note the same Y axis scales for **L** (a and c), and **S** (b and d). e) Normalized ICD values were read at 390 nm for **L** and 370 nm for **S**, where ICD values are normalized to that measured in the absence of lipids.

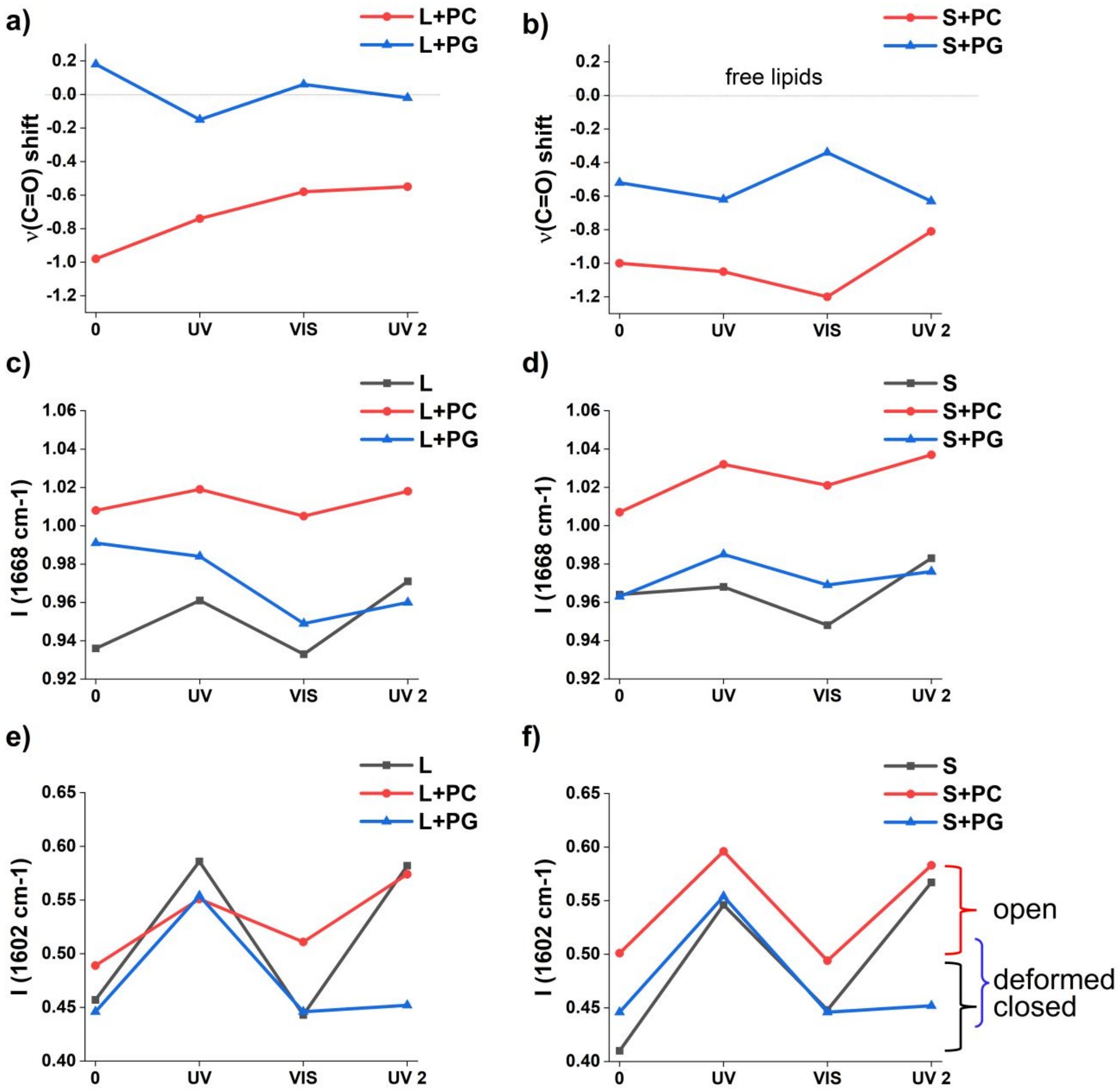

Figure S9. IR analysis of **1-GLFD** (**L** and **S**) upon UV-vis cycles in the presence and absence of PC or PG liposomes. Variations in lipid C=O vibration (a and b). Variations in peptide conformation (c and d). Variations in ring vibration (e and f). For more details see Infrared spectroscopy under Supporting text.

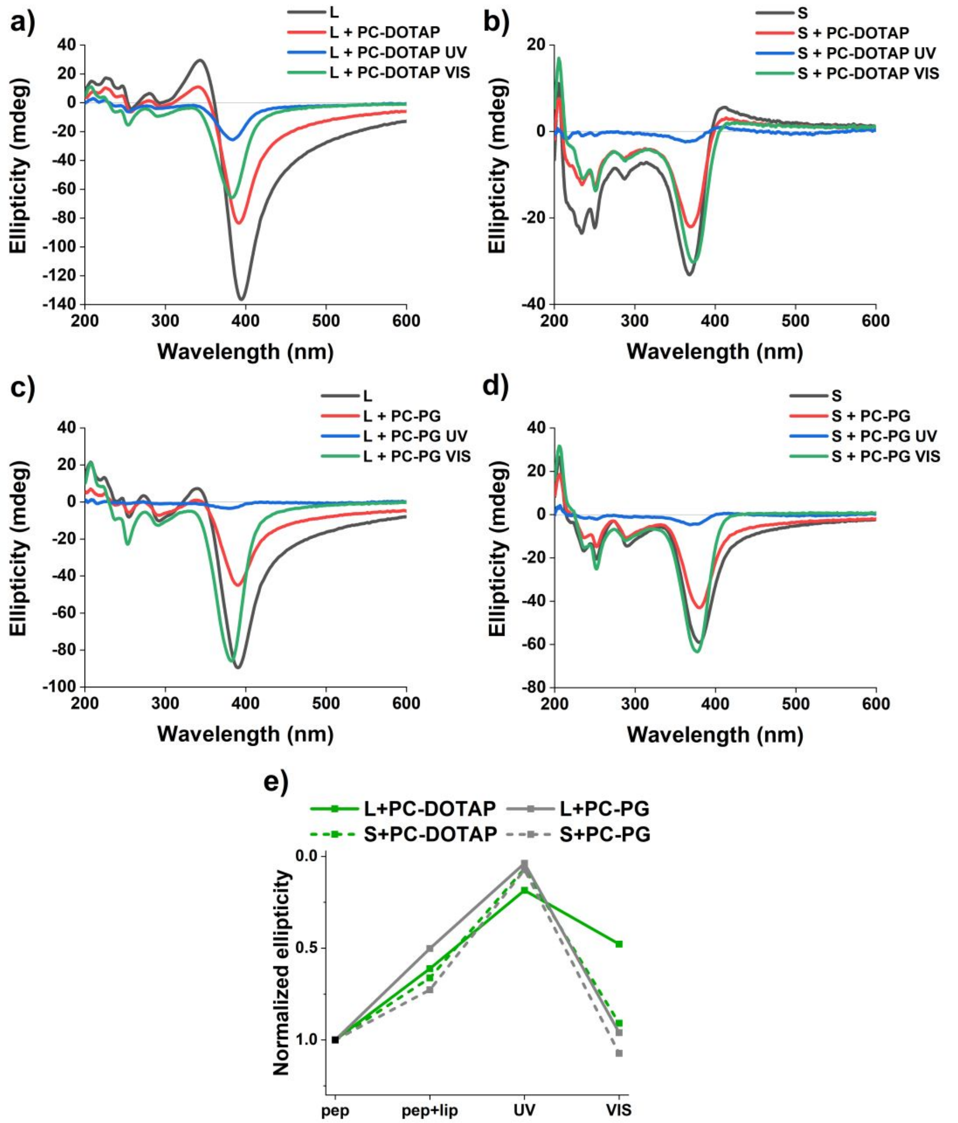

Figure S10. CD spectra of **L** and **S** with PC-PG and PC-DOTAP liposomes, and upon subsequent UV-vis irradiation. Spectra were recorded at 300 μM peptide and 0.635 mM lipid. Note the same Y axis scales for **L** (a and c), and **S** (b and d). e) Normalized ICD values read at 390 nm for **L** and 370 nm for **S**, where ICD values are normalized to that measured in the absence of lipids.

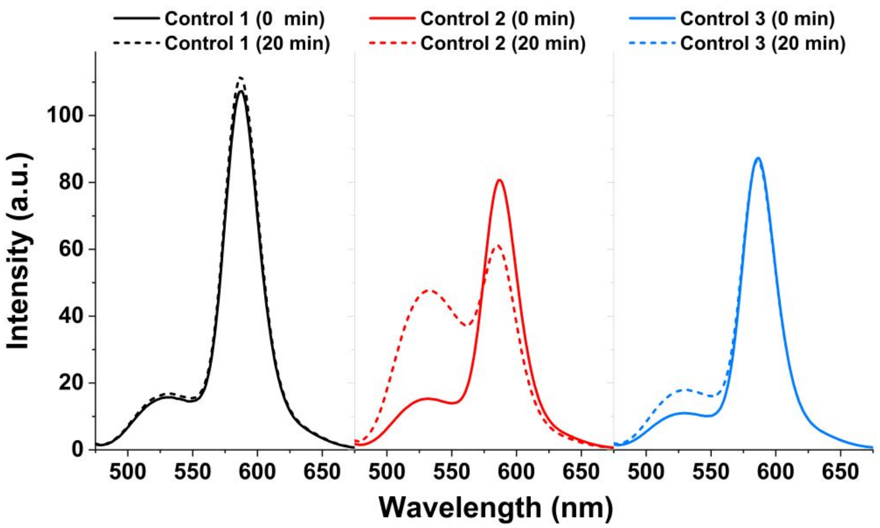

Figure S11. Fluorescence measurement of controls in 50 % wt of sucrose buffer. ***Control 1** = (NBD-PE : Rh-PE : PC) + PC, ***Control 2** = (NBD-PE : Rh-PE : PC-DOTAP) + PC-DOTAP, ***Control 3** = (NBD-PE : Rh-PE : PG) + PG.

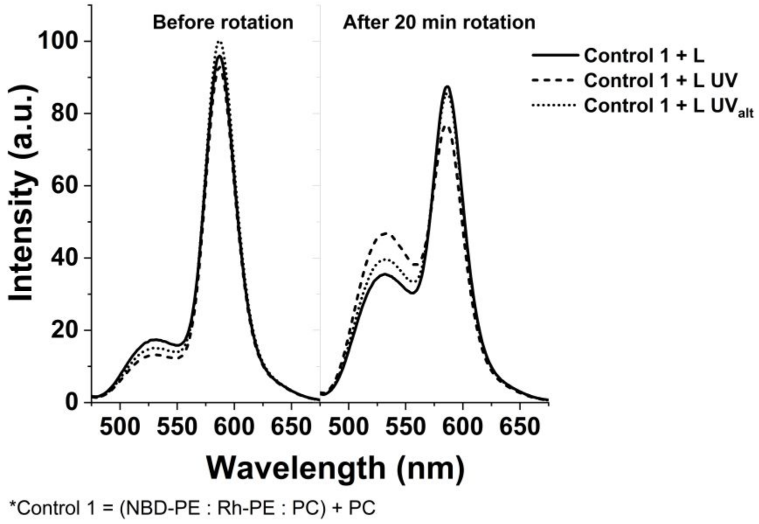

Figure S12. FRET efficiency of **L** with labelled (NBD-PE : Rh-PE : PC) and non-labelled (DOPC) liposomes in 50 % wt of sucrose buffer. The samples was rotated until 20 min with the shear rate ~3100 s^{-1}. ***Control** = (NBD-PE : Rh-PE : PC) + PC. **Control + L - UV** = single UV irradiation, **Control + L - UV_{alt}** = UV irradiation prior to sucrose insertion. The percentage of lipid mixing after 20 mins rotation for **Control + L** ~23%, **Control + L - UV** ~46% and **Control + L - UV_{alt}** is ~30%.

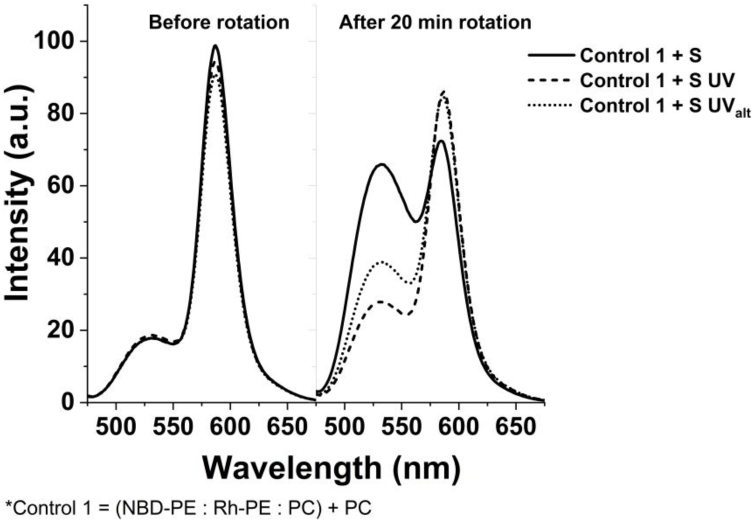

Figure S13. FRET efficiency of **S** with labelled (NBD-PE : Rh-PE : PC) and non-labelled (DOPC) liposomes in 50 % wt of sucrose buffer. The samples was rotated until 20 min with the shear rate ~3100 s^{-1}. ***Control** = (NBD-PE : Rh-PE : PC) + PC. **Control + S - UV** = single UV irradiation, **Control + S - UV_{alt}** = UV irradiation prior to sucrose insertion. The percentage of lipid mixing after 20 mins rotation for **Control + S** ~74%, **Control + S - UV** ~13% and **Control + S - UV_{alt}** is ~28%.

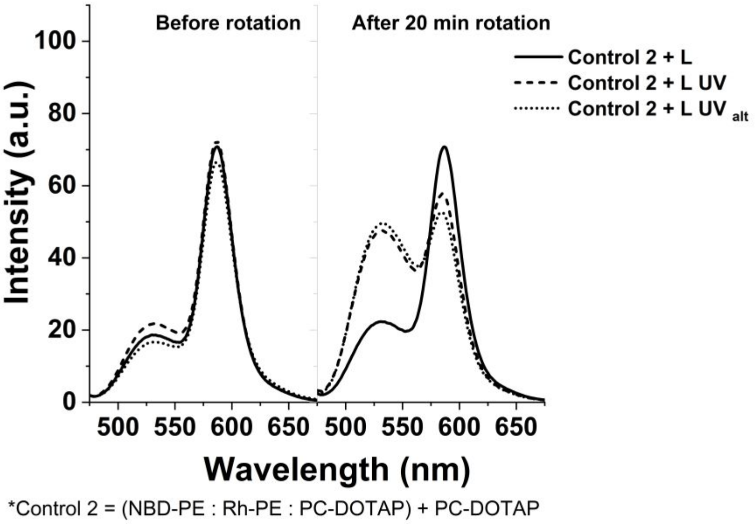

Figure S14. FRET efficiency of **L** with labelled (NBD-PE : Rh-PE : PC-DOTAP) and non-labelled (PC-DOTAP) liposomes in 50 % wt of sucrose buffer. The samples was rotated until 20 min with the shear rate ~3100 s^{-1}. ***Control** = (NBD-PE : Rh-PE : PC-DOTAP) + PC-DOTAP. **Control + L - UV** = single UV irradiation, **Control + L - UV_{alt}** = UV irradiation prior to sucrose insertion.

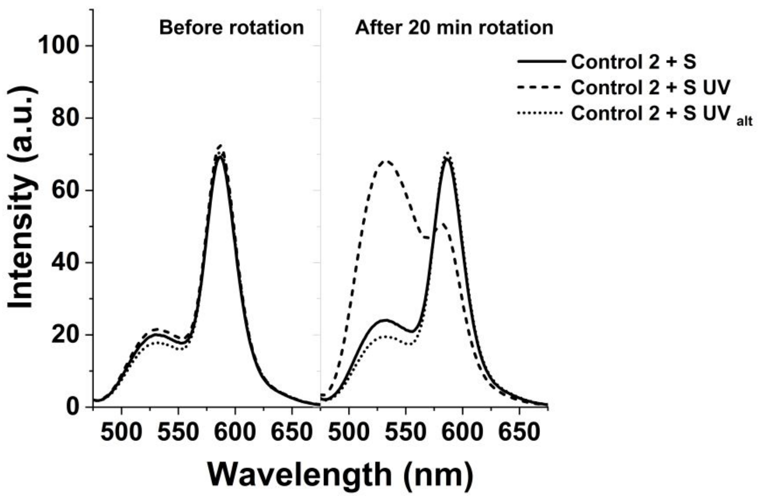

Figure S15. FRET efficiency of **S** with labelled (NBD-PE : Rh-PE : PC-DOTAP) and non-labelled (PC-DOTAP) liposomes in 50 % wt of sucrose buffer. The samples was rotated until 20 min with the shear rate ~3100 s^{-1}. ***Control** = (NBD-PE : Rh-PE : PC-DOTAP) + PC-DOTAP. **Control + S - UV** = single UV irradiation, **Control + S - UV_{alt}** = UV irradiation prior to sucrose insertion.

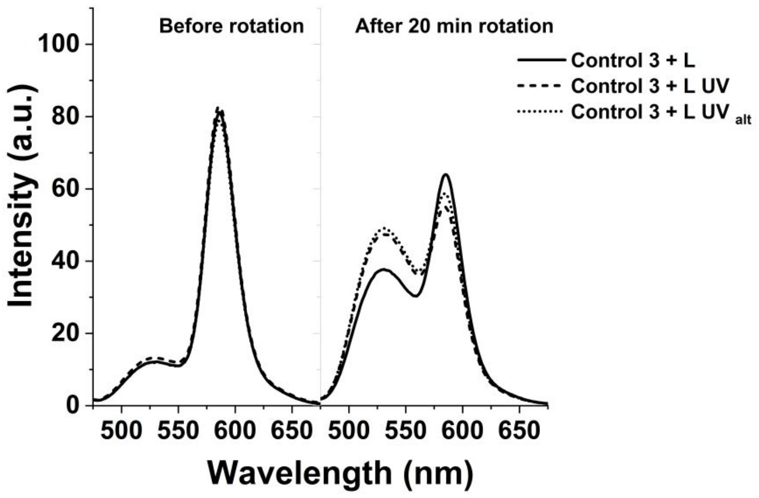

Figure S16. FRET efficiency of **L** with labelled (NBD-PE : Rh-PE : PG) and non-labelled (PG) liposomes in 50 % wt of sucrose buffer. The samples was rotated until 20 min with the shear rate ~3100 s^{-1}. ***Control** = (NBD-PE : Rh-PE : PG) + PG. **Control + L - UV** = single UV irradiation, **Control + L - UV_{alt}** = UV irradiation prior to sucrose insertion.

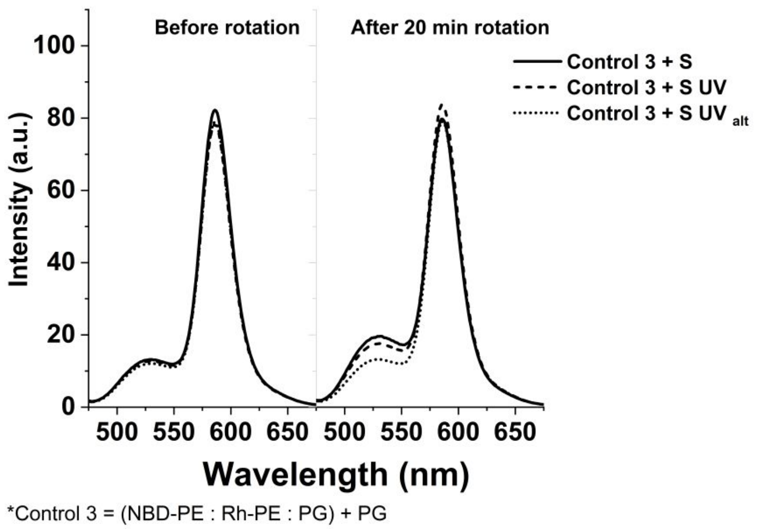

Figure S17. FRET efficiency of **S** with labelled (NBD-PE : Rh-PE : PG) and non-labelled (PG) liposomes in 50 % wt of sucrose buffer. The samples was rotated until 20 min with the shear rate ~3100 s^{-1}. ***Control** = (NBD-PE : Rh-PE : PG) + PG. **Control + S - UV** = single UV irradiation, **Control + S - UV_{alt}** = UV irradiation prior to sucrose insertion.

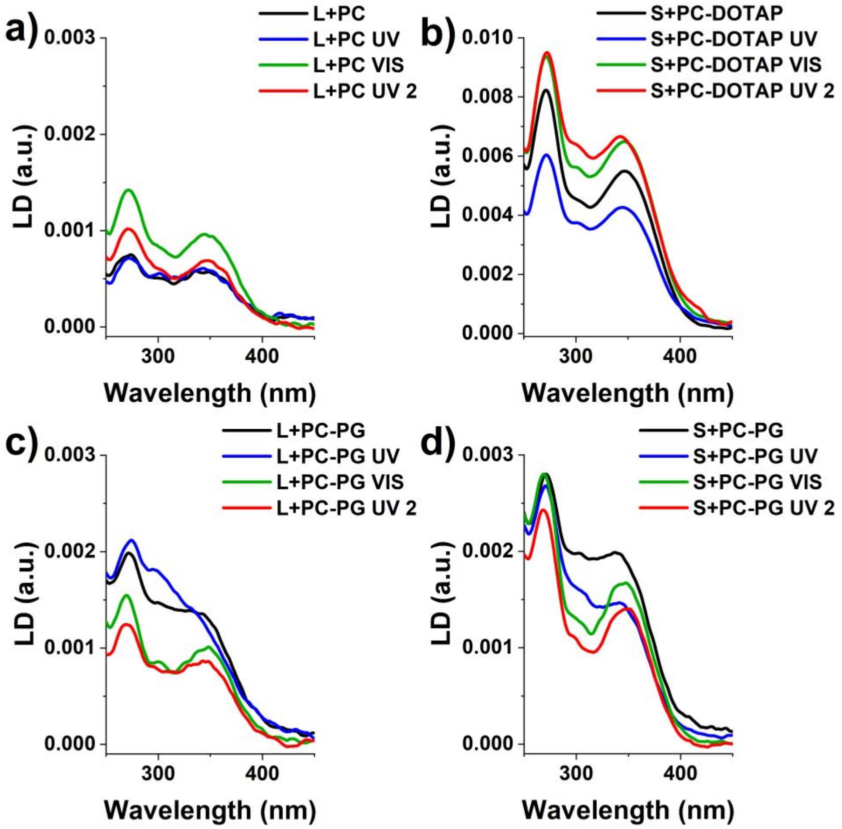

Figure S18. Flow-LD spectra of **1-GLFD** upon UV-vis irradiation in the presence of liposomes. Note that no LD peak is observed at ~500 nm for **1-(MC)-GLFD**, except for with PC-DOTAP (Figure 4). LD specta of **L** with PC and PC-PG (a and c). LD specta of **S** with PC-DOTAP and PC-PG (b and d).

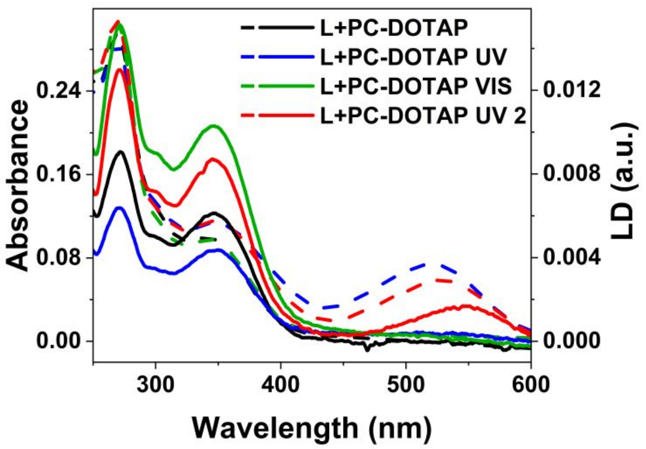

Figure S19. Flow-LD spectra and absorption spectra of **L** in the presence of PC-DOTAP liposomes during UV-vis irradiation cycles. Note that solid lines indicates LD signals and dashed lines represents absorbance peaks.

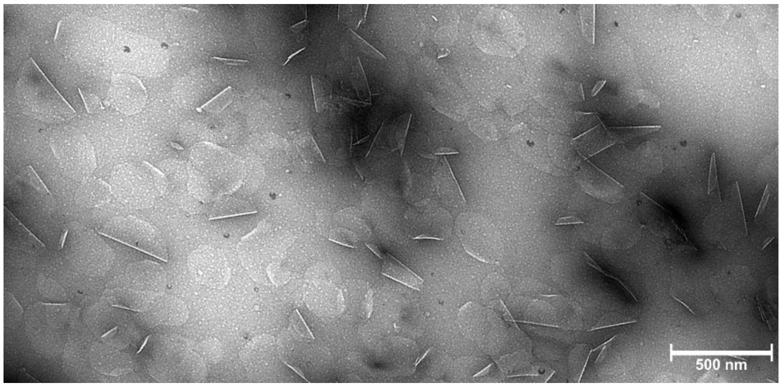

Figure S20. TEM image of **1-GLFD** nanofoils at 300μM peptide concentration in PBS buffer

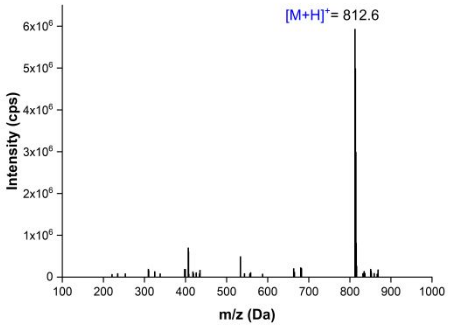

Figure S21. Mass spectrum of **SP-GLFD** peptide. m/z calculated for [$C_{42}H_{49}N_7O_{10}$] ([M+H]$^+$)= 812.36, observed ([M+H]$^+$)= 812.60.

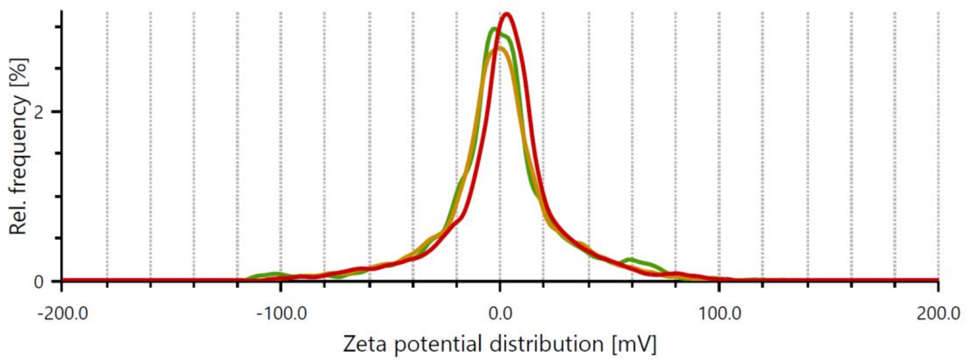

Figure S22. Zeta potential distribution of DOPC liposome (0.635 mM) in PBS buffer. The obtained zeta potential values are summarized in Table S3.

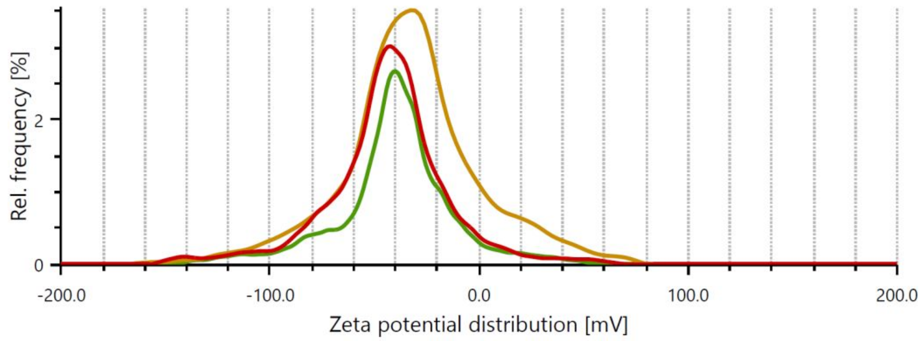

Figure S23. Zeta potential distribution of DOPG liposome (0.635 mM) in PBS buffer. The obtained zeta potential values are summarized in Table S3.

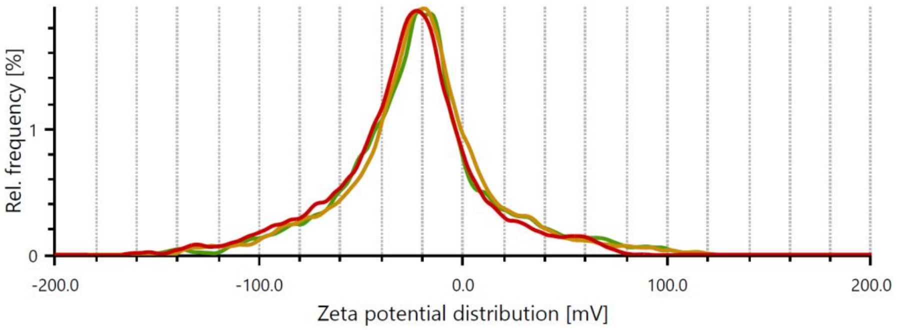

Figure S24. Zeta potential distribution of PC-PG liposome (0.635 mM) in PBS buffer. The obtained zeta potential values are summarized in Table S3.

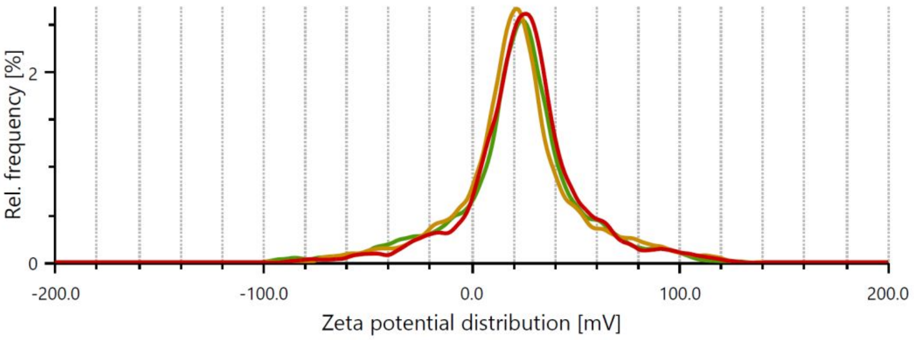

Figure S25. Zeta potential distribution of PC-DOTAP liposome (0.635 mM) in PBS buffer. The obtained zeta potential values are summarized in Table S3.

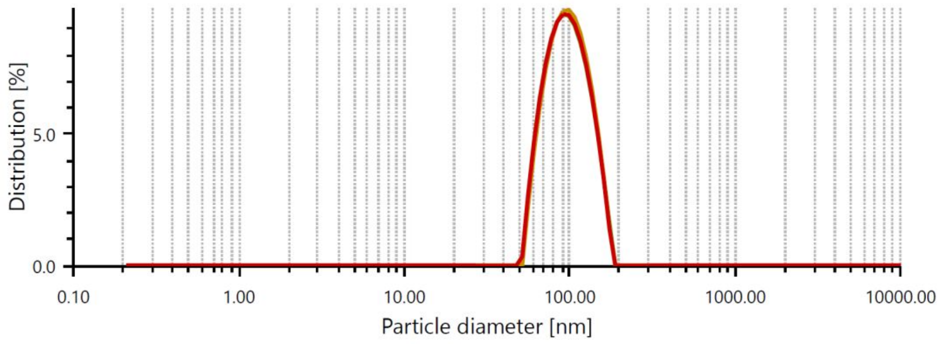

Figure S26. Particle size distribution (intensity vs. particle diameter) of DOPC liposome (0.635 mM) in PBS buffer. Mean hydrodynamic diameter (Dh) and polydispersity values are summarized in Table S4.

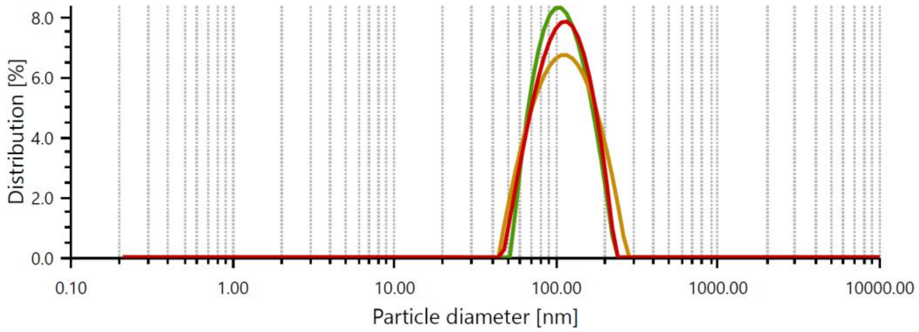

Figure S27. Particle size distribution (intensity vs. particle diameter) of DOPG liposome (0.635 mM) in PBS buffer. Mean hydrodynamic diameter (Dh) and polydispersity values are summarized in Table S4.

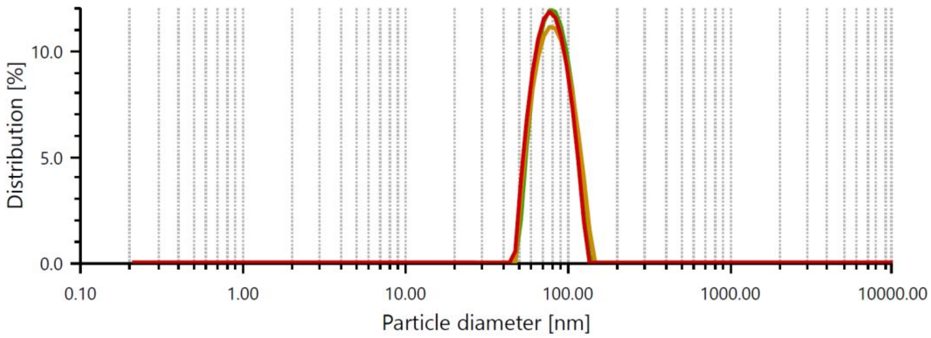

Figure S28. Particle size distribution (intensity vs. particle diameter) of PC-PG liposome (0.635 mM) in PBS buffer. Mean hydrodynamic diameter (Dh) and polydispersity values are summarized in Table S4.

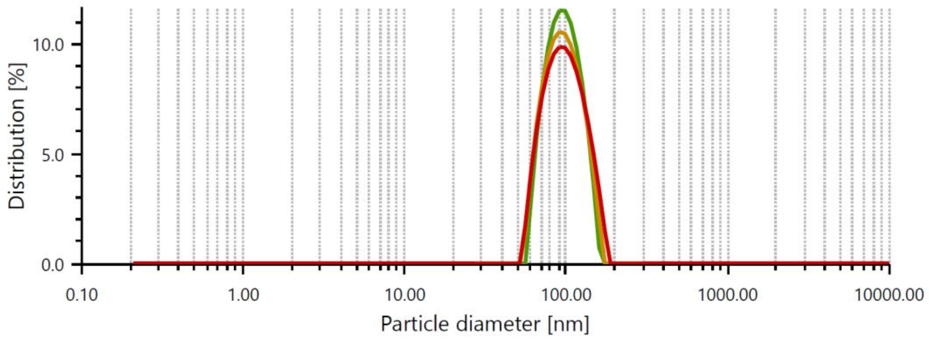

Figure S29. Particle size distribution (intensity vs. particle diameter) of PC-DOTAP liposome (0.635 mM) in PBS buffer. Mean hydrodynamic diameter (Dh) and polydispersity values are summarized in Table S4.

Table S1. Mean diameter, standard deviation and minimum and maximum size of **L** and **S** nanofoils measured from TEM images using ImageJ software.

	L (μm)	S (nm)
**Mean**	1.526	198.196
**SD**	0.65	72.606
**Min**	0.64	85.491
**Max**	3.506	379.67

Note: Data are based on measuring 100 individual nanofoils. * Min - Minimum size, Max - Maximum size.

Table S2. Absorbance peak maxima detected in UV-vis or LD absorbance spectra for **L** and **S** in the presence and absence of liposomes.

	PBS		PC		PC-PG		PC-DOTAP	
	0[1]	UV	0	UV	0	UV	0	UV
$S_{Abs\ max}$[a]	361	365, 520	345	350, 525	345	355, 525	350	360, 525
$L_{Abs\ max}$[a]	360	365, 520	345	355, 520	345	350, 525	350	360, 525
$S_{LD\ Abs\ max}$[b]	365	365	345	343	340	345	350	350
$L_{LD\ Abs\ max}$[b]	365	365	345	345	345	340	348	350, 546[2]

[a] **S / L** absorbance max in nm, [b] **S / L** LD absorbance max in nm, [1] Before UV irradiation, [2] Peak detected at 546 nm at UV2 irradiation.

Table S3. Zeta potential values obtained for the different liposome samples.

	Mean zeta potential [mV]	Distribution peak [mV]	Conductivity [mS/cm]	Electrophoretic Mobility [μm*cm/Vs]
**1. DOPC**				
**Mean value**	-1.7	0.4	22.103	-0.1250
**Standard deviation**	0.4	2.9	0.567	0.0314
**Rel. standard deviation**	25.13	807.74	2.56	25.13

**2. DOPG**				
**Mean value**	-44.7	-38.3	22.122	-3.3525
**Standard deviation**	1.2	5.0	0.504	0.0937
**Rel. standard deviation**	2.80	13.05	2.28	2.80
**3. PC-PG**				
**Mean value**	-20.2	-21.5	22.344	-1.5173
**Standard deviation**	0.3	1.9	0.575	0.0235
**Rel. standard deviation**	1.55	8.62	2.57	1.55
**4. PC-DOTAP**				
**Mean value**	20.3	24.0	22.374	1.5239
**Standard deviation**	0.8	2.4	0.652	0.0570
**Rel. standard deviation**	3.74	10.16	2.92	3.74

*Lipid concentration (0.635 mM)

Table S4. Mean hydrodynamic diameter (Dh) and polydispersity index of liposome samples.

	**Hydrodynamic diameter [nm]**	**Polydispersity index [%]**
**1. DOPC**		
**Mean value**	99.04	12.9
**Standard deviation**	1.25	2.5
**2. DOPG**		
**Mean value**	108.99	19.1
**Standard deviation**	1.44	2.2
**3. PC-PG**		
**Mean value**	85.07	6.8
**Standard deviation**	0.63	2.1
**4. PC-DOTAP**		
**Mean value**	99.52	10.2

**Standard deviation**	1.44	2.0

*Lipid concentration (0.635 mM)

For routine experiments, **SP-GLFD** powder was dissolved in methanol that was evaporated completely under a vacuum chamber to make a peptide dry film, which was finally hydrated with PBS and sonicated. Sonication time took from few mins up to 30 mins while sonication around 30 mins was found to be saturation or the dew point. To our surprise, we noticed significant changes in observed ICD values depending on the sonication time even though all samples exhibited ICD signals from -200 to -30 mdeg ~at 365 nm. Samples with short and long sonication time intervals of sonication showed ICD values of two characteristic ranges (Figure 1). Henceforth, we analysed the samples which were sonicated for less than 5 mins or for 30 mins. While some variation of the ICD values was observed because the aggregation process is irrepressibly influenced by the sonication, typical ICD values for the **L** and **S** form ranged from -200 to -100 mdeg and less than -90 to -30 mdeg, respectively. We concluded that upon sonication the orientation of the ring stacking is modified, which reduces the ICD signal intensity. Furthermore, the forming peptide assembly seems to be stable for several UV-vis cycles. The working concentration of the peptide was kept 300 µM for all the experiments, as significant and stable peptide self-assembly was observed at concentrations higher than 100 µM.

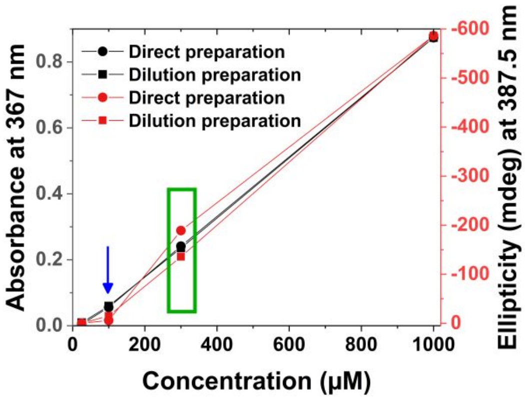

Absorbance and induced CD values of **SP-GLFD** at various concentrations (5, 25, 100, 300 and 1000 µM) prepared with direct and dilution methods. For the direct preparation, peptide (evaporated from methanolic stock) was dissolved directly at the indicated concentration upon addition of PBS and sonication for 30 mins. For the dilution preparation, the peptide was dissolved in PBS at 1 mM, and further diluted from this stock in PBS. The absorbance curve is perfect linear in the concentration range studied both methods, according to the linear proportionality of Abs vs conc. In contrast, the ICD curve shows a break-point at about 100 µM, which is indicative of assembly formation above this concentration.

Estimating size differences between S and L morphologies.
To address morphological differences between the two obtained forms, particle size measurements of **L** and **S** nanofoils from TEM images were performed by employing the ImageJ software, where the distinction of particles are based on the largest dimension of the observed nanofoils (Figure S2 and Table S1 in SI).[1–3]

SMALL-ANGLE X-RAY SCATTERING (SAXS)

The background-subtracted scattering curve of the **SP-GLFD** (5 mM) do not show any prominent peaks, the multilamellar stacking of self-assembled **SP-GLFD** bilayers can be precluded. In the shown double logarithmic plot, the curve starts with a power-law behavior, i.e. the intensity is proportional to q^{-2}. The exponent "-2" corresponds to the scattering of flat, layer- or disk-like particles, one dimension of which seems to be much smaller than the other two. The scattering of such systems can be described by the following functional form[4]:

$$I_{\text{flat}}(q) \approx \frac{A}{q^2} e^{-q^2 R_t^2}$$

where A is a scaling factor and R_t is the one-dimensional analogue of the radius of gyration, characteristic for the thickness of the flat particles. In the simplest case of homogeneous electron density, it is related to the actual thickness according to $R_t = T/\sqrt{12}$. Performing the least-squares fit of the above function to our data, the thickness of the layers was found to be 3.53 ± 0.09 nm. Assuming some stacking of the aromatic rings of spiropyran and their localization around the center, the electron density is somewhat higher around the bilayer center, and somewhat lower in the region of the **GLFD** peptide chains. This might be the reason that SAXS gives lower layer thickness, when compared to results from AFM and TEM.

INFRARED SPECTROSCOPY (ATR-FTIR)

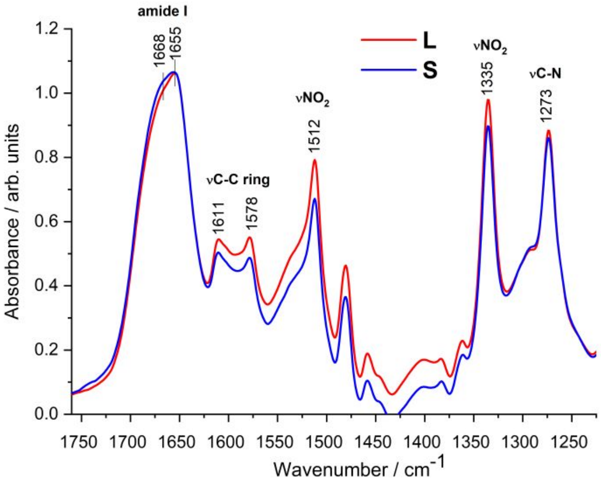

Representative ATR-IR spectra part of **L** and **S** forms. Spectra are normalized to the amide I band maximum at ~ 1555 cm^{-1}. Both spectra exhibit characteristic bands for both the ring, and the peptidic part. Specifically for the rings, C-C stretchings of the aromatic rings at 1611 and 1578 cm^{-1}, NO_2 stretchings at 1512 and 1335 cm^{-1} and C-N stretching at 1273 cm^{-1} can be assigned.[5] The **GLFD** motif shows a common amide I band, corresponding mainly to the C=O stretching of peptide bonds, with a maximum at around 1655 cm^{-1}. For both **L** and **S**, the amide I band profile is rather broad, with a definite shoulder at ~1668 cm^{-1}. This is indicative of two main peptide backbone populations with different H-bonding strength. The higher relative intensity of the shoulder band component in **S** is indicative of more oriented C=O dipole moments that is more oriented peptide chains in this form, which is in line with the proposed more compact packing of **S** over **L**. No notable differences were found between **L** and **S** related to the aromatic ring part.

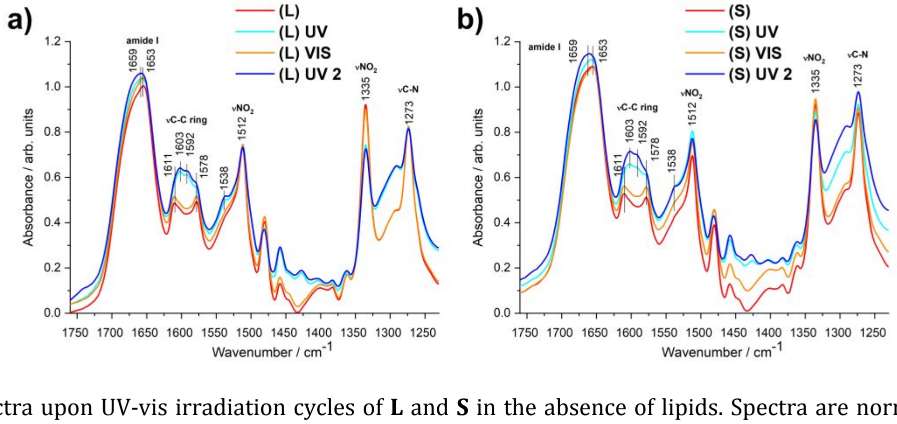

Parts of ATR-IR spectra upon UV-vis irradiation cycles of **L** and **S** in the absence of lipids. Spectra are normalized to the amide I band maximum at ~1555 cm^{-1} and shifted vertically for better visualization. Upon ring opening/closing, spectral changes in the C-C stretching band correspond to changes in the relative position of indoline and chromene moieties. Variations in the region (νC-C), and in the relative intensity of NO_2/C-N stretching bands are also observed. While a perfect reversibility was shown for **L** in two cycles, the fully open state was achieved only after the second UV irradiation for **S**. This is in line with the more compact packing of **S** compared to **L**. The amide I band of the peptide part is also affected upon ring opening, so that the band maximum shifts to

higher wavenumbers as the relative intensity of the band component at 1668 cm^{-1} increases. This indicates that ring movements upon opening induce changes in the orientation of **GLFD** motifs as well.

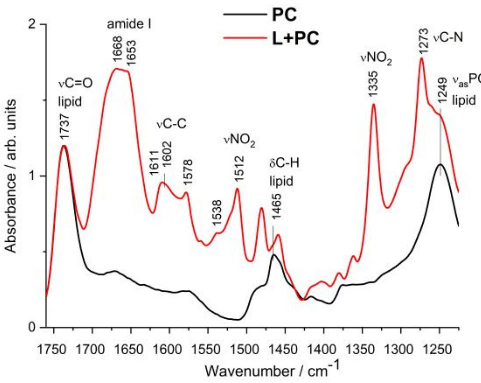

Representative ATR-IR spectra of a PC membrane in the presence and absence of **SP-GLPD**. Spectral regions of the applied PC and PG lipids are i) The symmetric CH_2 stretching vibration of the acyl chain (2588-2580 cm^{-1}). ii) the carbonyl stretching mode C=O of the lipid neck (1800-1680 cm^{-1}), iii) The phosphate head-group vibration region (1260-1000 cm^{-1}). The phosphate head-group vibrations are significantly masked by the C-N, O-C-N stretching and C-H deformation of SP, not allowing reliable analysis based on this band. Subtle changes were detected for the acyl C-H vibrations, however, there are also some spectral overlaps with peptide C-H vibrations. Most significant changes were observed for the lipid C=O vibrations, moreover, the peptide does not contribute to this spectrum region, so that this was used for detailed analysis. Based on the above considerations, three main spectral regions were selected for the analysis:

i) The shift in C=O stretching (νC=O) of lipids at 1800-1680 cm^{-1} reporting on the H-bonding pattern of the lipid neck region.
ii) The relative intensity of the amide I band component at 1668 cm^{-1}, reporting on the orientation/packing of the **GLFD** motif.
iii) The relative intensity of the band centered at ~1602 cm^{-1} assigned to ring C-C stretching reporting on the relative position of the chromene and the indoline moieties.

Variations in lipid C=O vibration. (Figure S9 a-b), Interaction with **L** affected the lipid carbonyl region of PC liposomes significantly indicating that **L** is bound down to the lipid neck region. Upon UV-vis cycles, the peptide remains lipid-bound, however, some lifting out of the bilayer is observed. Similar behavior was observed for **S** except of some more lipid perturbation upon the first UV-vis cycle. Similar binding mode could be concluded for **S** with PG, however, with smaller overall changes compared to PC. In contrast, shifts to the opposite direction were detected for **L** with PG lipids, which indicates a different binding mode compared tot he **S** form.

Variations in peptide conformation. (Figure S9 c-d), Interaction of **L** with both PC and PG affected the conformation/orientation of the peptide backbone (as reflected in the significantly shifted values prior irradiation) slightly more with PC. This PC-bound state was preserved upon UV-vis cycles, however, small perturbations were detected related to ring opening/closing. In contrast, the peptidic part turned towards the lipid-free state after the first UV irradiation. **S** with PC behaved similarly as **L**. By contrast, no definite changes were detected for **S** with PG.

Variations in ring vibration. (Figure S9 e-f), Changes in SP ring vibrations showed good reversibility for both **L** and **S** in the absence of lipids The reversible nature was preserved in the presence of membranes except the second UV irradiation with PG. The latter suggest hampered ring opening in the second cycle in this case. It should be noted, however, that binding to PC induced noticeable ring deformation (as reflected in the significantly shifted band intensity ratio prior irradiation), resulting in a deformed, stressed, still closed, ring system with changes in the angle between the planes of the indoline and chromene parts as turning to some extent towards the open form. Further on, the open state adopted after the second UV irradiation with PC seems to converge to that resembling lipid-unbound **1-GLFD**. In contrast to PG, peptide interaction with PC could likely facilitate ring opening in the lipid-bound state, particularly for **S**.

NMR SPECTROSCOPY

The conversion of spiropyran to merocyanine form was also monitored by NMR spectroscopy. Both **L** and **S** forms were prepared for the experiments. Characteristic ^1H signals were assigned to the spiropyran and merocyanine forms enabling the differentiation of both forms in the spectra. Sample were prepared the same way in PBS as in other investigations, characterized, and prior the NMR measurement 10 V/V% D_2O was added. The colourless/slightly yellow solutions contained significant majority of the closed spiropyran form, 96% for **S** and 85% for **L**. After 5 mins UV irradiation, the pink solutions showed different ratios of the spiropyran and merocyanine forms. In the **L**-system, a more facilitated conversion was found giving high proportions of the merocyanine form: 77% which remained 72% after further 12 h storage in the dark. By applying the same conditions for the **S**-system much lower amounts were observed: 22% of the merocyanine form after UV irradiation and 27% after storage in dark. The **L** form was prepared also in a PBS solution made with 100% D_2O as a solvent. This sample gave similar ratios as in H_2O/D_2O 90:10. In the

initial sample, spiropyran form was the majority with 89%. The UV irradiation for 5 minutes causes the efficient conversion to the merocyanine form (90%). The broadening of the signals and low signal intensities in the spectra suggest self-association of the peptides, mainly on Visible light. Because of this, the correlations were very weak in the 2D spectra and did not enable complete assignment of the monomers and define long-range NOE-correlations in the assemblies.

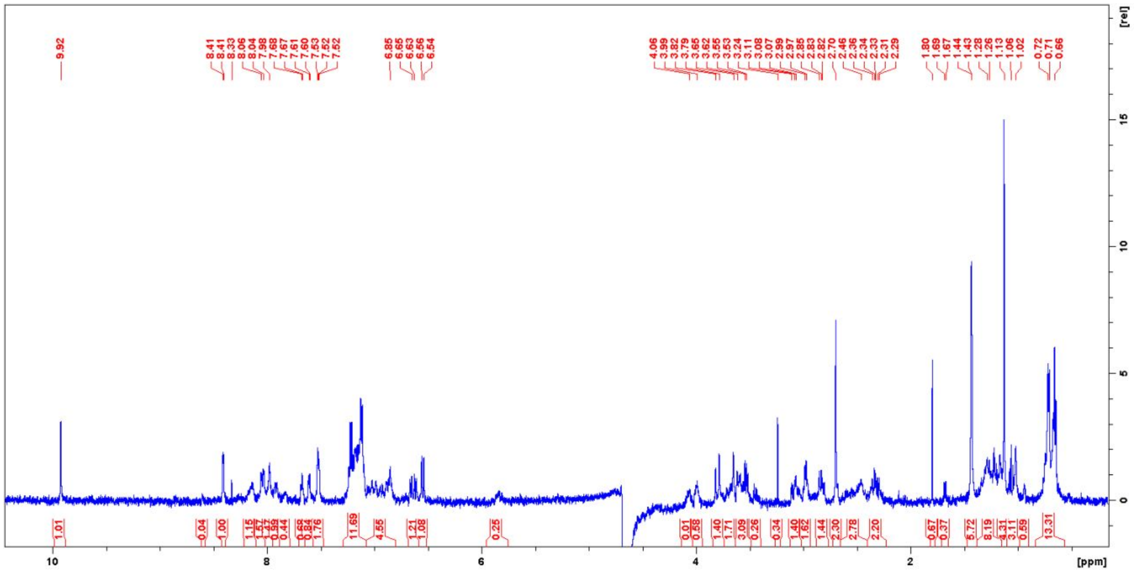

^{1}H NMR spectrum of **S** (initial sample, stored on Visible light) in PBS (H_2O/D_2O 90:10).

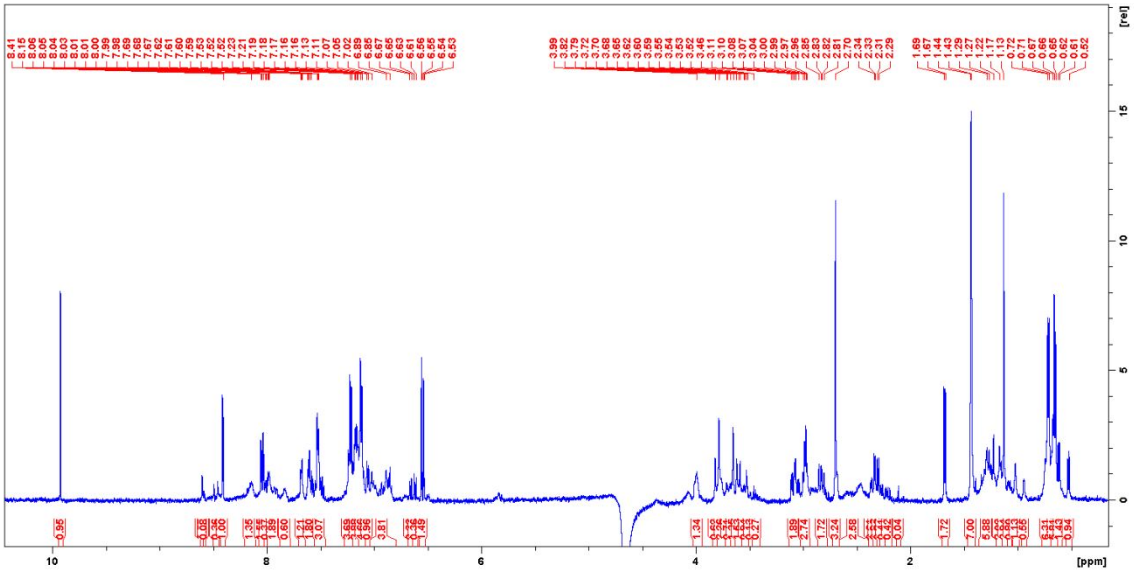

^{1}H NMR spectrum of **S** (sample after 5 min UV irradiation) in PBS (H_2O/D_2O 90:10).

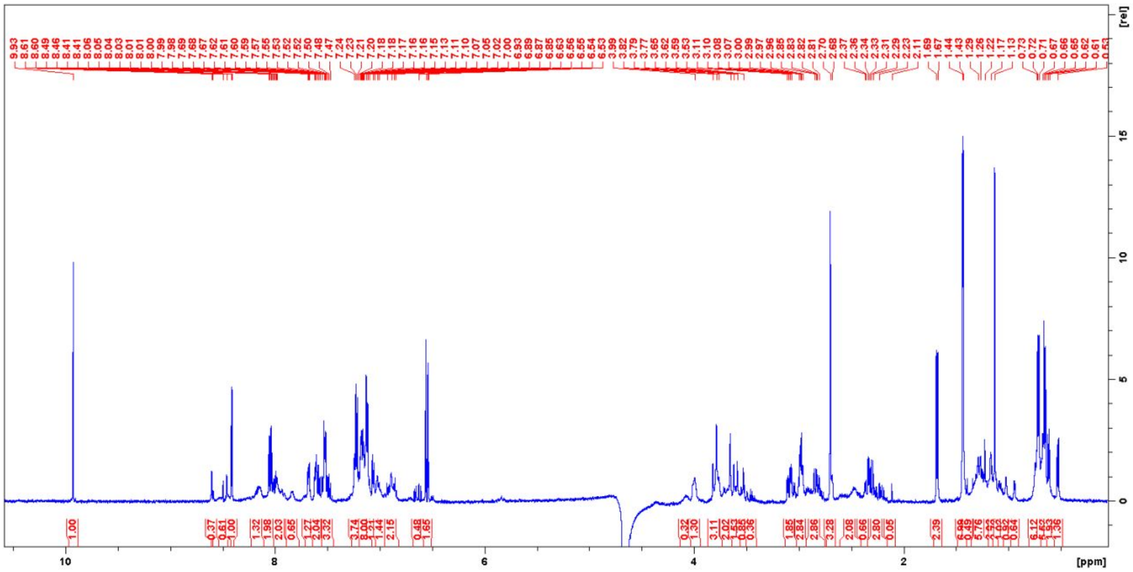

^{1}H NMR spectrum of **S** (sample after 5 min UV irradiation and stored for additional 12h in dark) in PBS (H_2O/D_2O 90:10).

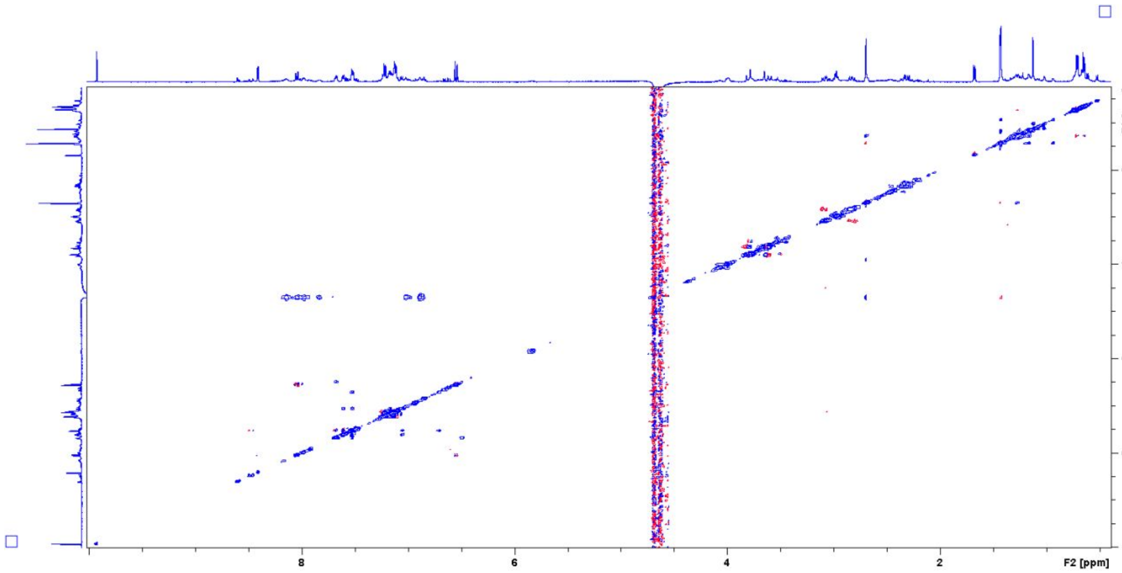

ROESY NMR spectrum of **S** (sample after 5 min UV irradiation) in PBS (H_2O/D_2O 90:10).

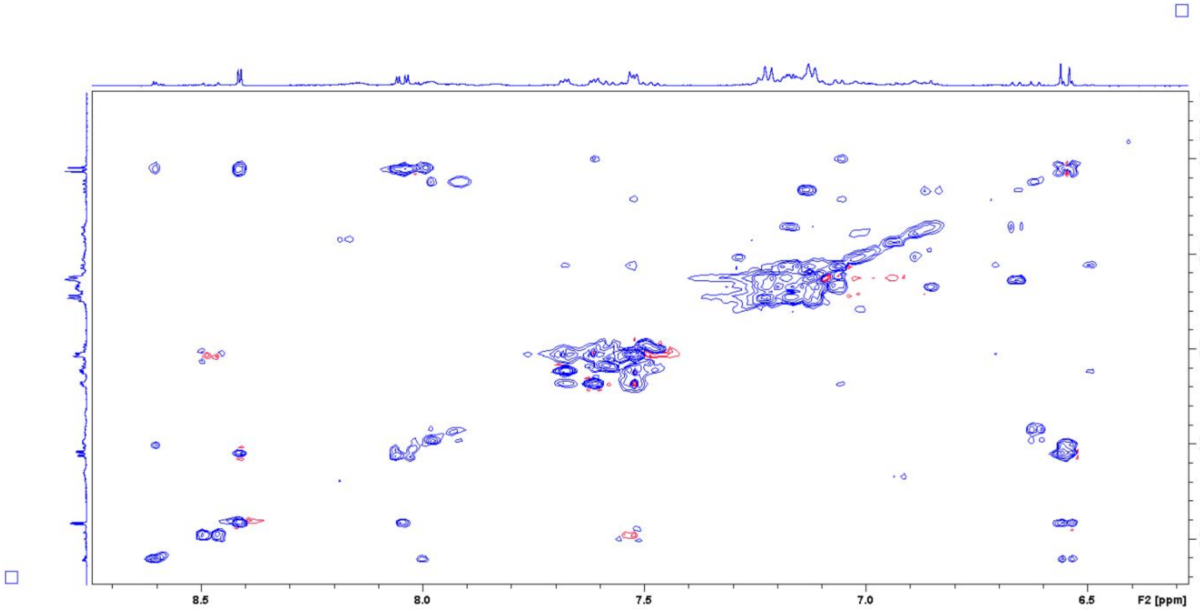

TOCSY NMR spectrum of **S** (sample after 5 min UV irradiation) in PBS (H_2O/D_2O 90:10).

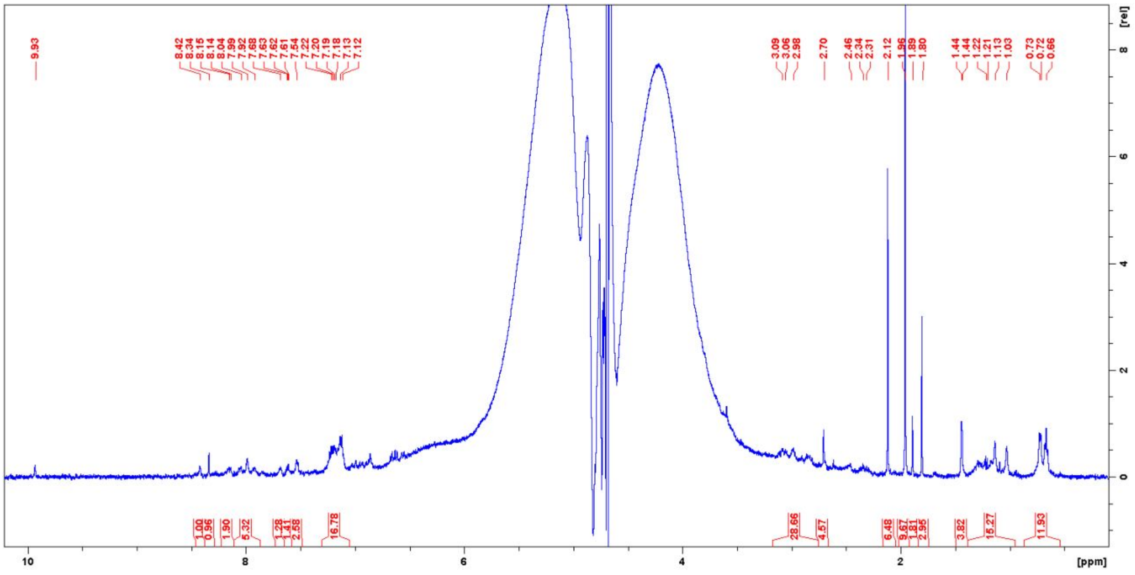

^1H NMR spectrum of **L** (initial sample, stored on Visible light) in PBS (H_2O/D_2O 90:10).

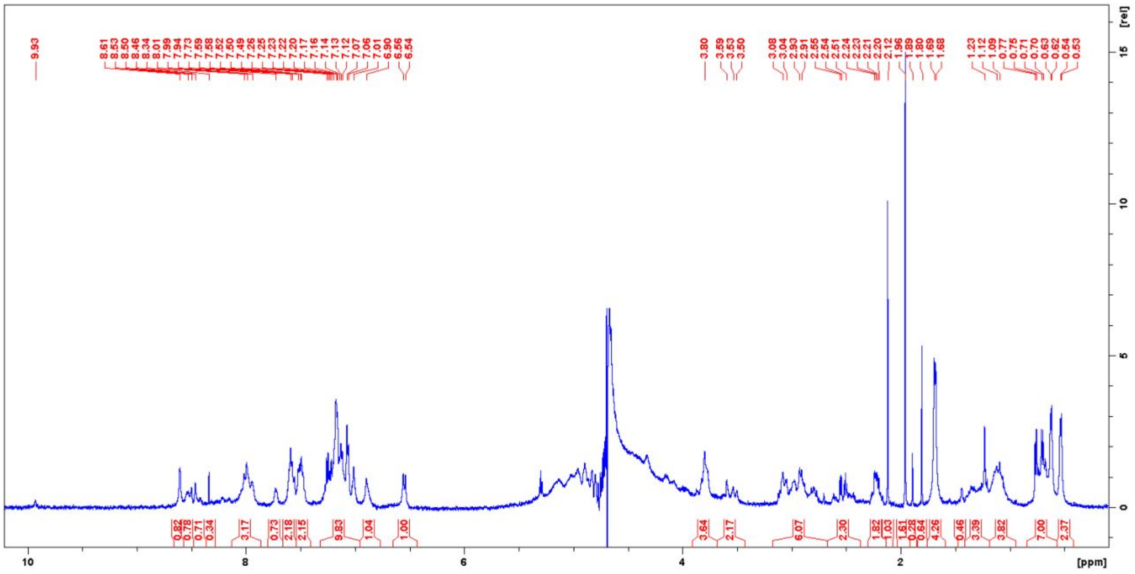

^1H NMR spectrum of **L** (sample after 5 mins UV irradiation) in PBS (H_2O/D_2O 90:10).

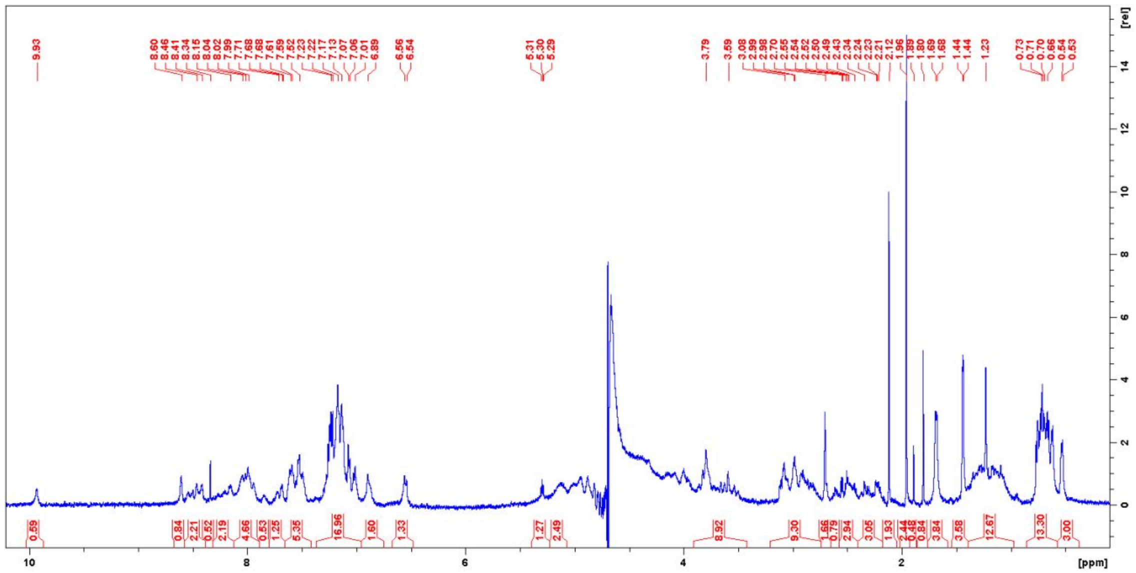

^{1}H NMR spectrum of **L** (sample after 5 mins UV irradiation and stored for additional 12 h in dark) in PBS (H_2O/D_2O 90:10).

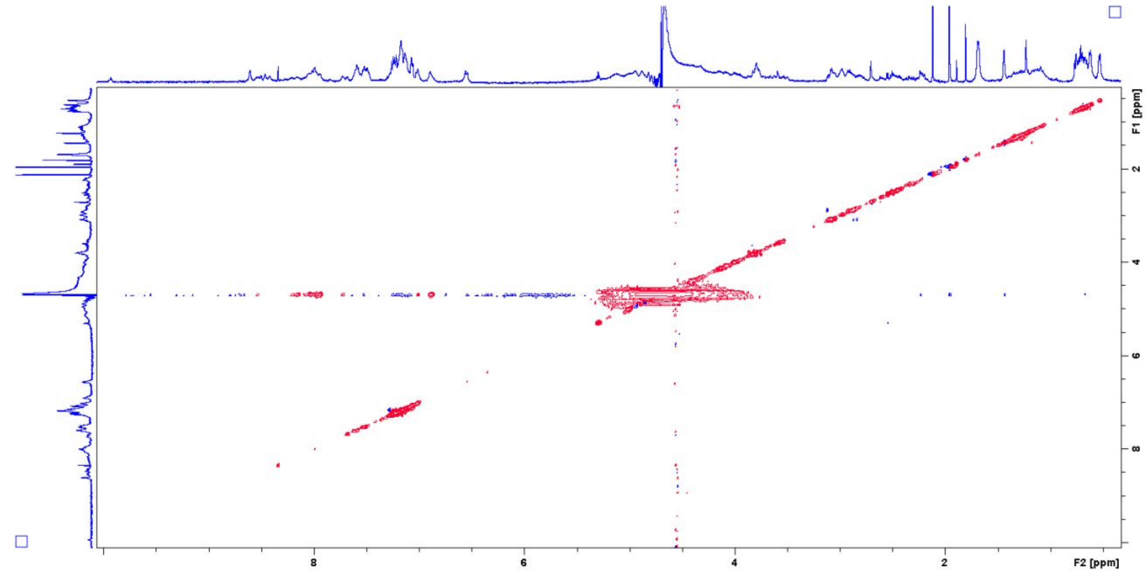

ROESY NMR spectrum of **L** (sample after 5 mins UV irradiation) in PBS (H_2O/D_2O 90:10).

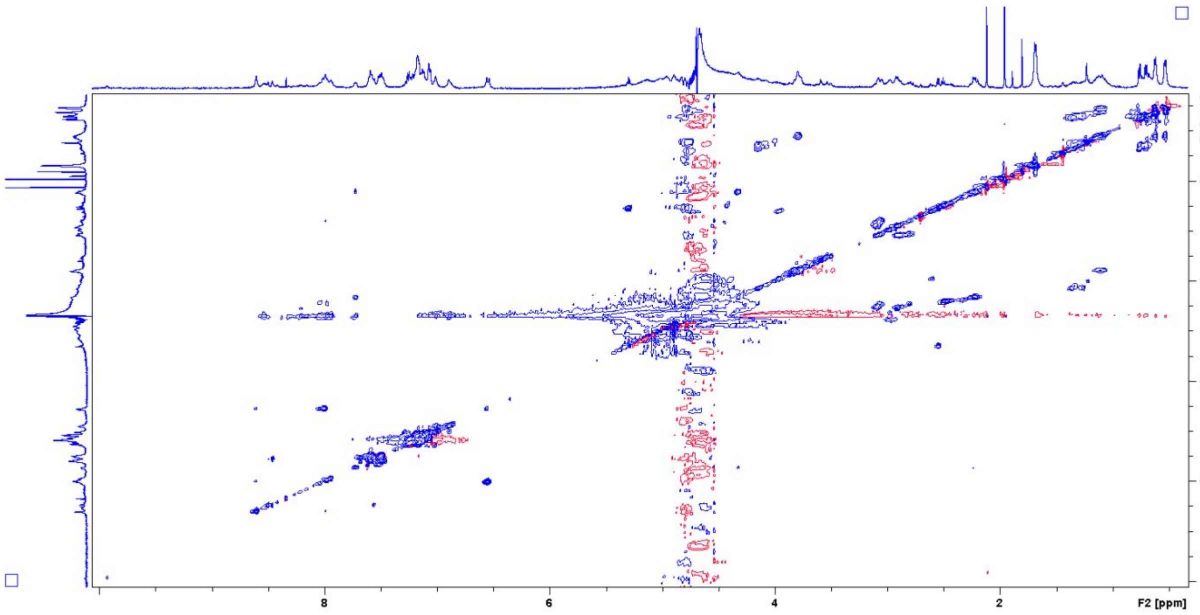

TOCSY NMR spectrum of **L** (sample after 5 mins UV irradiation) in PBS (H_2O/D_2O 90:10).

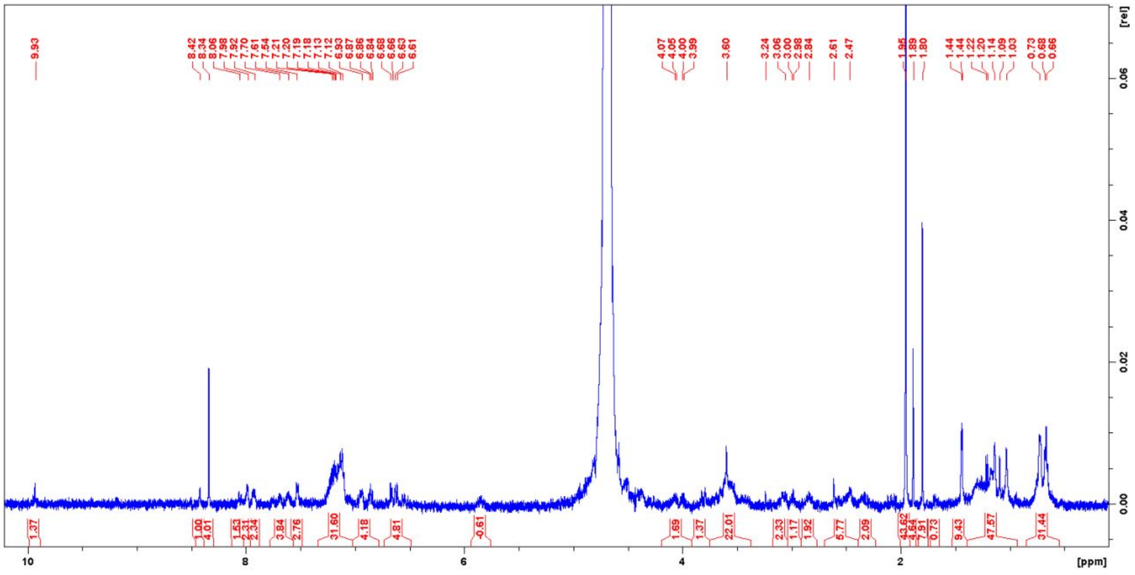

^{1}H NMR spectrum of **L** (initial sample, stored on Visible light) in PBS (D_2O 100%).

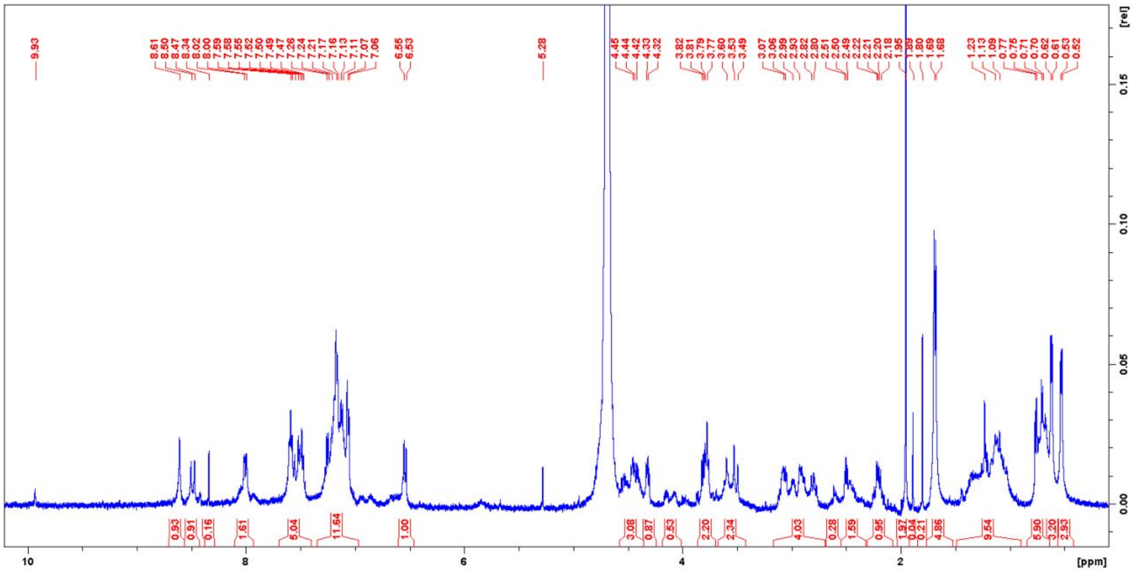

^{1}H NMR spectrum of **L** (sample after 5 mins UV irradiation) in PBS (D_2O 100%).

REFERENCES

(1) Igathinathane, C.; Pordesimo, L. O.; Columbus, E. P.; Batchelor, W. D.; Methuku, S. R. Shape Identification and Particles Size Distribution from Basic Shape Parameters Using ImageJ. *Comput. Electron. Agric.* **2008**, *63* (2), 168–182. https://doi.org/10.1016/j.compag.2008.02.007.

(2) Karbasi, S.; Alizadeh, Z. M. Effects of Multi-Wall Carbon Nanotubes on Structural and Mechanical Properties of Poly(3-Hydroxybutyrate)/Chitosan Electrospun Scaffolds for Cartilage Tissue Engineering. *Bull. Mater. Sci.* **2017**, *40* (6), 1247–1253.

https://doi.org/10.1007/s12034-017-1479-9.
(3) Ramaraj, H.; Madiga, J.; Elangovan, H.; Haridoss, P.; Sharma, C. P. Homogenization for Dispersion and Reduction in Length of Carbon Nanotubes. *Trans. Indian Inst. Met.* **2017**, *70* (10), 2629–2639. https://doi.org/10.1007/s12666-017-1123-9.
(4) Porod, G. General {{Theory}}. In *Small {{Angle X-ray Scattering}}*; Glatter, O., Kratky, O., Eds.; Academic Press: London, 1982; pp 17–51.
(5) Delgado-Macuil, R.; Rojas-López, M.; Gayou, V. L.; Orduña-Díaz, A.; Díaz-Reyes, J. ATR Spectroscopy Applied to Photochromic Polymer Analysis. *Mater. Charact.* **2007**, *58* (8-9 SPEC. ISS.), 771–775. https://doi.org/10.1016/j.matchar.2006.12.003.
